# Finger Millet [*Eleusine coracana* (L.) Gaertn.] Improvement: Current Status and Future Interventions of Whole Genome Sequence

**DOI:** 10.3389/fpls.2018.01054

**Published:** 2018-07-23

**Authors:** S. Antony Ceasar, T. Maharajan, T. P. Ajeesh Krishna, M. Ramakrishnan, G. Victor Roch, Lakkakula Satish, Savarimuthu Ignacimuthu

**Affiliations:** ^1^Division of Plant Biotechnology, Entomology Research Institute, Loyola College Chennai, India; ^2^Functional Genomics and Plant Molecular Imaging Lab, University of Liege, Liege, Belgium; ^3^Department of Biotechnology Engineering, Ben-Gurion University of the Negev, Beersheba, Israel; ^4^The Jacob Blaustein Institutes for Desert Research, Ben-Gurion University of the Negev, Beersheba, Israel

**Keywords:** finger millet, whole genome sequence (WGS), millets, nutrient transport, genomic resources

## Abstract

The whole genome sequence (WGS) of the much awaited, nutrient rich and climate resilient crop, finger millet (*Eleusine coracana* (L.) Gaertn.) has been released recently. While possessing superior mineral nutrients and excellent shelf life as compared to other major cereals, multiploidy nature of the genome and relatively small plantation acreage in less developed countries hampered the genome sequencing of finger millet, disposing it as one of the lastly sequenced genomes in cereals. The genomic information available for this crop is very little when compared to other major cereals like rice, maize and barley. As a result, only a limited number of genetic and genomic studies has been undertaken for the improvement of this crop. Finger millet is known especially for its superior calcium content, but the high-throughput studies are yet to be performed to understand the mechanisms behind calcium transport and grain filling. The WGS of finger millet is expected to help to understand this and other important molecular mechanisms in finger millet, which may be harnessed for the nutrient fortification of other cereals. In this review, we discuss various efforts made so far on the improvement of finger millet including genetic improvement, transcriptome analysis, mapping of quantitative trait loci (QTLs) for traits, etc. We also discuss the pitfalls of modern genetic studies and provide insights for accelerating the finger millet improvement with the interventions of WGS in near future. Advanced genetic and genomic studies aided by WGS may help to improve the finger millet, which will be helpful to strengthen the nutritional security in addition to food security in the developing countries of Asia and Africa.

## Introduction

Finger millet (*Eleusine coracana* (L.) Gaertn.) is an allotetraploid (2n = 4X = 36, AABB) belonging to the Family Poaceae and the genus *Eleusine.* The genome size of finger millet is 1,593 Mb and is a self-pollinated crop (Goron and Raizada, 2015). It is an annual herbaceous cereal crop widely grown and consumed by poor people in Africa and Asia. It contains rich amounts of protein, mineral nutrient as compared to other major cereals like wheat, rice, and sorghum (Gupta et al., 2017; Sharma et al., 2017). Finger millet is well known for its exceptionally high calcium (Ca) content having about 0.34% in whole seeds as compared with 0.01–0.06% in most other cereals (Kumar et al., 2016; Gupta et al., 2017). The seeds are abundant source of dietary fiber, iron, essential amino acids viz., isoleucine, leucine, methionine, phenylalanine, pytates and trypsin inhibitory factors, and are also gluten-free (Chandra et al., 2016; Sood et al., 2016). Finger millet also has many health-promoting benefits such as hypoglycemic, hypocholesterolemic and anti-ulcerative effects (Chethan and Malleshi, 2007). The grain is used as flour in the preparation of cakes, bread and other pastry products, and also serves as a beneficial food for infants (Mgonja et al., 2007; Ceasar and Ignacimuthu, 2011). The seeds can be stored for more than 5 years without insect damage which makes it a most valuable crop in drought-prone areas of Africa (Latha et al., 2005). According to estimates, about 3.5 billion people were at the risk of Ca deficiency in 2011 and about 90% of these people were living in Africa and Asia (Kumssa et al., 2015). Crops such as rice and wheat can provide food security, but finger millet has nutritional properties superior to that of rice and wheat, so it has been proposed to help in strengthening the nutritional security in the developing countries of Asia and Africa (Puranik et al., 2017).

**FIGURE 1:**
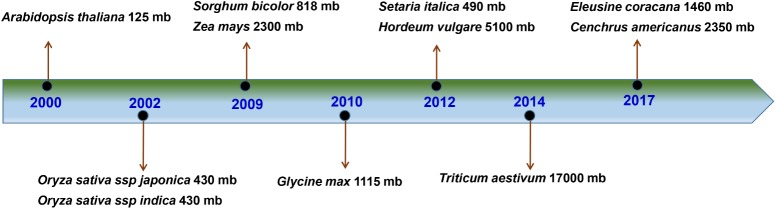
Milestones in the genome sequencing of cereals. The scale shows the year of release of genome sequence for key cereal. The scientific name and genome size are indicated for each cereal. Source: www.plabipd.de

Establishment of genetic and genomic resources is a crucial step forward in improving the crop plants for specific traits. Rapid developments in the tools like Illumina sequencing in recent years have accelerated the whole genome and transcriptome sequencing in several plants (Bolger et al., 2014). As a result, the whole genome sequence (WGS) has become available for model plants and many cereals, even with more complex genomes^1^. The whole genome sequencing of finger millet has been delayed as compared to other major cereals, leaving it as one of the lastly sequenced genomes among cereals (**Figure 1**). For e.g., the first draft genome for rice was released in 2005 (International Rice Genome Sequencing, 2005) with the completion of annotation in 2013 (Kawahara et al., 2013). Foxtail millet is the only millet to have its WGS released with complete annotations till date. The WGS of 2 different foxtail millet genotypes were released in 2012 (Bennetzen et al., 2012; Zhang et al., 2012). However, the first draft genome of finger millet was released only recently (Hittalmani et al., 2017), more than a decade after the release of rice draft genome (**Figure 1**). As a result, only a few genetic and genomic studies have been performed in finger millet and the high resolution genetic and genomics studies are lagging behind due to the lack of WGS for finger millet. The recently released draft genome is expected to serve as a major resource for the accelerated studies for the improvement of finger millet in near future.

In this article, we review the details of various studies undertaken to improve the finger millet including genetic improvement, identification of quantitative trait loci (QTLs) for key traits, gene characterization and transcriptome analysis. We have collected the details on genomic resources and literature on finger millet from public database like NCBI, PubMed and major publishers’ sites such as, Springer, Elsevier, etc., ranging from 1975 to till date. We discuss the past works, analyze shortfalls and provide insights on the interventions of WGS in aiding finger millet improvement in future.

## Finger Millet Germplasm and Production

More than 28,041 finger millet germplasms are available in various organizations worldwide. Of this, the National Bureau of Plant Genetic Resources (NBPGR), India, has 10,507 germplasm and the International Crop Research Institute for the Semi-Arid Tropics (ICRISAT), India has 5957 germplasms. Other institutes like Kenya Agricultural Research Institute (KARI), Kenya (2875), Institute of Biodiversity Conservation (IBC), Ethiopia (2156), USDA Agricultural Research Service (USDA-ARS), United States (1452) and Serere Agricultural and Animal Production Research Institute (SAARI), Uganda have a reasonable collection of germplasm (Dwivedi et al., 2012; Goron and Raizada, 2015; Saha et al., 2016; Gupta et al., 2017).

**FIGURE 2:**
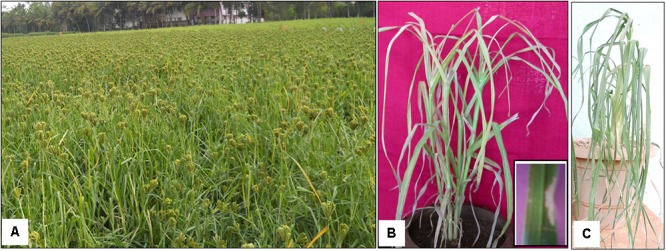
Finger millet growth and stresses. **(A)** Photograph showing the cultivation of finger millet in Coimbatore district in Tamil Nadu state of South India; **(B)** 2 months old finger millet affected by leaf blast disease in glass house condition, the insert image shows the magnification of leaf infection by the fungus; **(C)** 2 months old finger millet affected by drought stress. Images **(B,C)** were obtained from the experiments conducted by SAC and LS.

Finger millet is majorly grown in the semi-arid tropics of Asia and Africa. In Asia, finger millet is mostly grown in the Southern states of India which provide favorable growth conditions (**Figure 2A**). Among the millets, finger millet ranks fourth on a global scale of production next to sorghum, pearl millet (*Cenchrus americanus*), and foxtail millet (*Setaria italica*) (Upadhyaya et al., 2007). Around 4.5 million tons of finger millet are produced worldwide every year. Africa produce 2.5 million tons and India produces 1.2 million tons annually. Finger millet accounts for about 85% all millets produced in India and is cultivated over 1.19 million hectares in India according to a recent report (Sakamma et al., 2018).

As the increase in population and industrialization throughout the world reduced the availability of agricultural land, by the end of 2050, the world is expected to face a severe food demand (Gupta et al., 2017). To overcome such a situation, there is an urgent need to increase the production of cereals like finger millet, which has to be increased up to 4.5 t ha^-1^ by 2025 (Borlaug, 2002). Finger millet will be an ideal crop for climate resilient agriculture due to its adaptation in semi-arid tropics which are characterized by unpredicted weather and erratic rainfall. So it will be a good cereal for harsh climate due to global warming. Increasing the finger millet production will make this high nutritional food available for the poor people of developing nations and will help to attain nutritional security.

## Constraints of Finger Millet Production

Finger millet production is severely affected by both biotic and abiotic stresses (Saha et al., 2016) (**Figure 2B**). Fungal blast is a major disease affecting growth and yield of finger millet (Kumar and Kumar, 2011). Blast diseases are caused by an ascomycete fungus *Magnaporthe oryzae* (anamorph: *Pyricularia grisea*) (Singh and Kumar, 2010). The fungus mostly infects young leaf and causes leaf blast, whereas under highly favorable conditions, neck and finger blasts are also formed at flowering (Babu et al., 2013). Ekwamu (1991) reported that the head blast significantly reduced the spikelet length, grain weight, number of grains per head and grain yield. The blast fungus enters and causes the breakdown of parenchymatous, sclerenchymatous, and vascular tissues of the neck region, thereby inhibiting the flow of nutrients into the grains (Rath and Mishra, 1975). Subsequently, grain formation is partially or totally inhibited (Rath and Mishra, 1975; Ekwamu, 1991). The infected spikelets were shorter than healthy spikelets, which affects the grain formation. Eventually, the high seed infection reduced the seed germination in the field (Gashaw et al., 2014). The average loss owing to the blast has been reported to be around 28–36% per hectare (Nagaraja et al., 2007) and according to an earlier study, the yield losses could be as high as 80–90% per hectare (Rao, 1990).

Major abiotic stresses such as deficiencies of nutrients [nitrogen (N), phosphorus (P), and zinc (Zn)], drought, and salinity also seem to affect the growth and yield of finger millet (Yamunarani et al., 2016; Ramakrishnan et al., 2017; Maharajan et al., 2018). According to a recent study, N deficiency decreased the tiller number in finger millet (Goron et al., 2015). Low P stress also affected the growth and biomass of finger millet seedlings in glass house conditions (Ramakrishnan et al., 2017). Zn deficiency resulted in stunted growth, delayed seed maturity, appearance of chlorosis, shortened internodes and petioles, and malformed leaves (Yamunarani et al., 2016). Drought is also one of the major abiotic constraints of finger millet production (**Figure 2C**). Parvathi et al. (2013) studied the effect of drought stress on the expression of candidate genes in genotype GPU-28. Drought stress caused wilting and leaf rolling and resulted in the reduction of leaf solute potential and chlorophyll content with the induction of many drought stress responsive genes when compared to control condition (Parvathi et al., 2013). Salinity also reduced the water content, plant height, leaf expansion, finger length and width, grain weight, and delayed the flowering (Anjaneyulu et al., 2014). Seedlings of finger millet genotype GPU-28 exposed to salinity stress, PEG and oxidative stress showed significant reduction in plant growth and shoot and root biomass (Parvathi and Nataraja, 2017).

Nutrient deficiency may be one of the major abiotic stresses affecting the finger millet production in the future. For example, the demand for fertilizers like N is expected to rise steadily, during the forecast period, from 8.8% in 2017 and reaching 9.5% in 2018 (Food and Agriculture Organization of the United States [FAO], 2015). In 2018, the global potential balance of P fertilizer is expected to rise from 6.4 to 8.5% of total demand (Khabarov and Obersteiner, 2017). Developing plants with improved P-use efficiency has been considered as essential to reduce the P fertilizer usage (Baker et al., 2015; Ceasar, 2018). Based on the Food and Agriculture Organization (FAO) analysis, N and P demands may also affect the production of finger millet in future. This is an important issue since crops like finger millet are majorly grown by resource poor farmers in low input agricultural systems of Asia and Africa who cannot afford to buy expensive fertilizers (Thilakarathna and Raizada, 2015). Breeding of finger millet with genetic and genomic studies aided by recently released WGS may be helpful to develop new genotypes that are tolerant to multiple nutrient stresses.

## Current Status of Genomic Resources Available for Finger Millet

**FIGURE 3:**
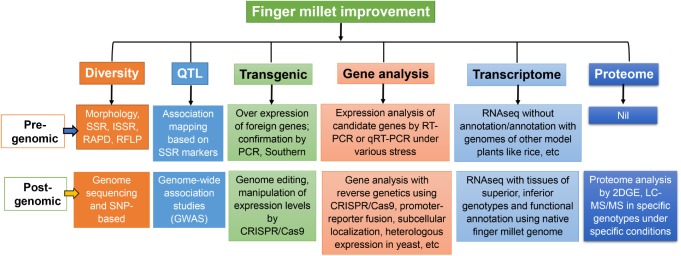
Finger millet improvement in the pre-genomic and post genomic eras. Various approaches used for the improvement of finger millet like, diversity analysis, QTL mapping, transgenic modification, gene analysis are indicated. The use of advance tools for the improvement of finger millet in post-genomic era based on the whole genome sequence are indicated.

The genomic resources available for finger millet are limited as compared with other major cereals which hampers the further improvement of this crop (Saha et al., 2016). The details of various genomic resources available for finger millet, rice, barley and maize at NCBI are listed in **Table 1**. For e.g., only a few expressed sequence tags (ESTs) are available in finger millet compared to those of rice, maize and barley. The finger millet has only 1934 ESTs which is almost 100 times lower than that of maize and rice and 50 times lower than that of barley (**Table 1**). No complete gene and Unigene sequence has yet been reported for finger millet. Several genome assemblies are available for other cereals as compared to just only one for finger millet (ASM218045v1). Similarly, limited number of proteins were reported for finger millet when compared to 3 other major cereals (**Table 1**). Till date, no single nucleotide polymorphism (SNP) has been developed in finger millet genome. The recently released WGS of finger millet will be helpful to build all these resources in the coming years to accelerate finger millet research at all spheres of studies (**Figure 3**). Finger millet also has a limited number of transcriptome sequences obtained from a few stress conditions and for grain Ca content (**Table 2**). Few efforts were made to sequence the transcriptome of specific genotypes subjected to various stresses like drought, saline and blast. However, the validation of sequence reads information and further characterization of key genes have not yet been accomplished in most of these studies and have simply been submitted as raw reads (**Table 2**). The recently released WGS of finger millet is expected to serve as a major resource for making several of these resources and for further studies. For e.g., the RNAseq reads can be validated using the WGS of finger millet to find key genes involved in each process (**Figure 3**).

**Table 1:** Details on genomic and proteomic resources available for finger millet, rice, maize, and barley.

Name of the sequence/resource	Finger millet (*Eleusine coracana*)	Rice (*Oryza sativa*)	Maize (*Zea mays*)	Barley (*Hordeum vulgare*)
EST	1,934	1,281,057	2,023,541	840,300
Gene	0	97,446	78,018	707
Unigene	0	74,892	61,577	20,224
Genome assembly	01	26	17	09
Clone	0	172,025	1,145,013	0
Nucleotide	1,095	771,335	1,059,632	3,536,399
SNP	0	13,218,961	58,915,360	0
Protein	554	1,324,842	332,077	69,529
Protein cluster	0	15,559	94	77
Protein structure	3	210	330	142

*Source, NCBI; Date of collection, 5th March 2018.*

**Table 2:** Details on genome and transcriptome sequences reported for finger millet under various experimental conditions.

S. No	Name of the genotype	Type of sequence	Property/trait	NICBI Accession no.
1	PR-202	Genome assembly	Drought stress	PRJDB5606
2	^∗∗^	Metagenome	Blast disease	PRJNA383952
3	KNE796	Whole genome and transcriptome	Crop improvement	PRJNA377606
4	ML-365	Transcriptome	Moisture stressed	PRJNA339512
5	ML-365	Whole genome	Drought stress	PRJNA318349
6	KNE796		High throughput marker development	PRJNA317618
7	GPU-28	Transcriptome	Drought stress	PRJNA282860
8	MR-1	smallRNA analysis	Drought stress	PRJNA277250
9	CO 12 and Trichy 1	Transcriptome	Salinity stress	PRJNA236733
10	^∗∗^	Transcriptome	Water deficit	PRJNA229808
11	GPU-28	Transcriptome	Drought stress	PRJNA282859
12	^∗∗^	Transcriptome	Drought stress	PRJNA282578
13	^∗∗^	Transcriptome	Blast disease	PRJNA268401
14	GPU-1 and GPU-45	Transcriptome	Calcium content	PRJNA236796

*Source, NCBI; Date of collection, 5th March 2018. ^∗∗^Details not provided.*

## Whole Genome Sequence

The much awaited WGS of finger millet genotype ML-365 (a drought tolerant and blast disease resistant genotype with good cooking qualities) was obtained recently using Illumina and Sequencing by Oligonucleotide Ligation and Detection SOLiD sequencing technologies (Hittalmani et al., 2017). Around 45 Gb paired end and 21 Gb mate-pair data were generated. The genome assembly consisted of 525,759 scaffolds (>200 bp) with N50 length of 23.73 Kb, and the average scaffold length of 2275 bp (Hittalmani et al., 2017). The transcriptome was also successfully sequenced and assembled in this study for well-watered (WW) (53,300 unigenes) and low moisture stressed (LMS) (100,046 unigenes) plants of genotype ML-365. Among the unigenes assembled, nearly 64% were functionally annotated with Viridiplantae protein sequences using UniProt database. The differential gene expression analysis revealed that 2,267 unigenes were specific to WW, 12,893 were specific to LMS and 111,096 unigenes were found in both WW and LMS conditions. Further, protein domain analysis predicted several functional proteins in the expressed genes. Plant transcription factors (TFs) were mined by protein-protein homology modeling and a total of 11,125 genes were predicted to have homology with 56 TF families. Overall, 2866 drought responsive genes were associated with major TF families across 19 Pfam domains. About 1766 genes were identified as R-genes for various diseases and 330 genes were found to be involved in calcium transport and accumulation (Hittalmani et al., 2017). The WGS of finger millet was found to have greater co-linearity with foxtail millet and rice as associated to other Poaceae species. This study also revealed that the genome sizes of *E. coracana* subspecies *coracana* and *E. coracana* subspecies *africana* were relatively similar (Hittalmani et al., 2017). This may be due to the fact that finger millet was domesticated from *E. coracana* subspecies *africana* (Dida et al., 2008).

Hatakeyama et al. (2018) reported the WGS and assembly of finger millet genotype PR-202 (IC: 479099) using a novel polyploidy genome assembly workflow. Their initial analysis identified the genome size of finger millet as 1.5 Gb and the assembled genome was 1189 Mb which was estimated to cover 78.2% genome. The whole genome of genotype PR-202 consisted of 2387 scaffolds with the N50 value of 905.318 Kb having maximum sequence length of 5 Mb. The FASTA file format of final scaffolds and annotation are publicly available at NCBI (BioSample number: SAMD00076255). Overall, 62,348 genes were identified by this study, nearly 91% genes were functionally annotated and 96.5% were found to be single-copy genes. The NCBI BLAST analysis identified that a total of 57,913 genes was duplicated with more than two copies in the genome of PR-202 (Hatakeyama et al., 2018).

The availability of WGSs of ML-365 and PR-202 can be used effectively for further studies, such as SNP identification, next-generation sequencing (NGS)-based allele discovery, linkage and association map construction, identification of candidate genes for agronomically important traits, functional characterization of candidate genes using reverse genetic approaches and marker-assisted breeding programs (**Figure 3**).

## *In Vitro* Studies: a Prerequisite for Genetic Transformation

Establishment of an efficient *in vitro* regeneration protocol is a vital prerequisite for the transformation and regeneration of cereals (Shrawat and Lörz, 2006). *In vtiro* culture has been considered essential for finger millet improvement (Yemets et al., 2013). Several reports are available for the *in vitro* regneration of finger millet using various explants in different genotypes (Supplementary Table S1). The types of explants used include shoot tip (Eapen and George, 1990; Ceasar and Ignacimuthu, 2008, 2011), leaf sheath fragments (Eapen and George, 1990; Gupta et al., 2001), embryogenic seed (Kothari et al., 2004; Latha et al., 2005; Sharma et al., 2011; Babu et al., 2012), mature and immature embryos (Kumar et al., 2011), undeveloped inflorescence (Eapen and George, 1990; Kumar et al., 2011), root mesocotyl (Mohanty et al., 1985), and leaf-base segments (Rangan, 1976; Mohanty et al., 1985) (Supplementary Table S1). Satish et al. (2015) made an attempt to regenerate finger millet through direct organogenesis using shoot apical meristem. The same group also developed an efficient *in vitro* regeneration protocol for indirect organogenesis in four Indian genotypes (CO (Ra)-14, GPU-25, Try-1, and Piyur-2) using plant growth regulators and polyamine compounds like spermidine (Satish et al., 2016b). Use of seaweed liquid extracts seem to promote the somatic embryogenesis and regeneration in the same genotypes of finger millet (Satish et al., 2016a). Recently, yet another direct plant regeneration protocol was developed in three genotypes [CO 9, CO (Ra)-14 and GPU-28] of finger millet (Babu et al., 2018).

Shoot apex explant is an ideal material for efficient *in vitro* regeneration owing to its easy availability, accessibility, rapid regeneration of multiple shoots, and easier to handle when compared with other explants (Arockiasamy and Ignacimuthu, 2007; Ceasar and Ignacimuthu, 2008; Dey et al., 2012). Shoot apex was used in the past for finger millet regeneration. The direct plant organogenesis is also an effective method to produce more multiple shoots with less somoclonal variation in a short time as it minimizes the culture duration for callus formation, sub-culturing cycles and quicker regeneration of transgenic plants following transformation (Satish et al., 2015). There is no report available till date for *in vitro* regeneration through anther culture, protoplast and protoplasmic fusion in finger millet. Development of anther culture in finger millet could help to develop the haploid lines. The development of protoplasmic fusion may also help to improve the hybrid variety of finger millet. The WGS may also be utilized for clustered regularly interspaced short palindromic repeats (CRISPR) and CRISPR associated protein 9 (Cas9)-mediated genome editing in finger millet for which protoplast mediated transformation looks very effective. So the establishment of protoplast based regeneration in finger millet may be helpful to achieve these tasks taking finger millet research into next and higher level.

## Genetic Improvement of Finger Millet

**FIGURE 4:**
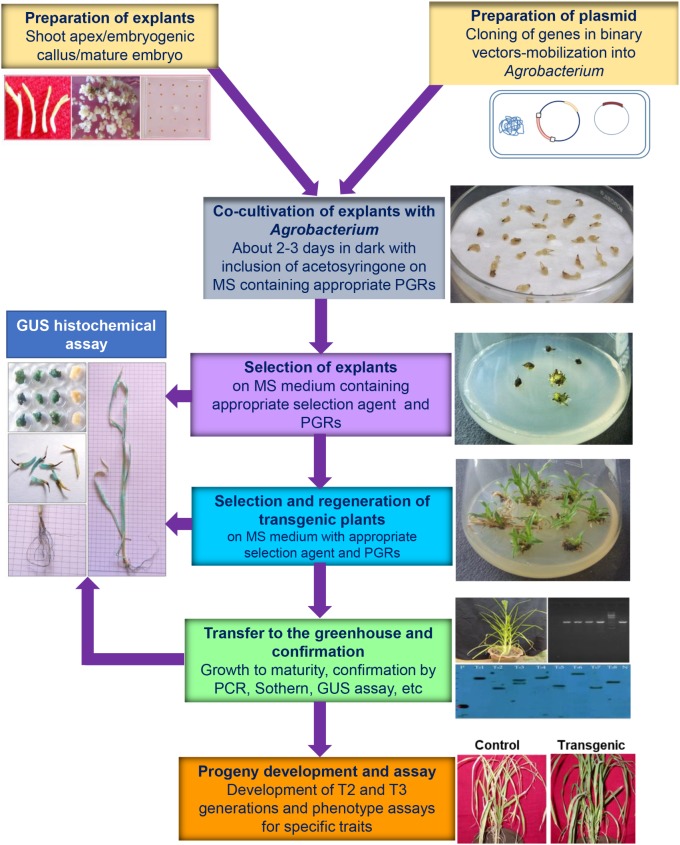
General protocol used in the *Agrobacterium*-mediated transformation of finger millet. The stepwise protocol used in the *Agrobacterium*-mediated transformation is illustrated with respective figures. The photographs were obtained from the works performed in the labs of SI, SAC, and LS. The bio-assay photograph was obtained in the transgenic finger millet resistant to leaf blast disease and control plants by SAC.

Genetic improvement of finger millet has been lagging behind when compared to the efforts made for other major cereals. Improved genetic transformation of millets including finger millet has been considered essential to improve the nutritional quality, and resistance to abiotic and biotic stresses (Ceasar and Ignacimuthu, 2009). Gupta et al. (2001) initiated the preliminary work on transformation of finger millet using biolistic method for comparing the efficiency of five gene promoters [*cauliflower mosaic virus 35s* (*CaMV35S)/rice actin gene promoter ActI/maize ubiquitin (UqI)/ribulose-1,5-biohosphate carboxylase small subunit gene promoter(RbcS)/Flaveria trinervia β-glucuronidase* (*FtuidA*) on the expression of the *β-glucuronidase* (*GUS*)] reporter gene. Following this, a few studies reported on the optimization of transformation conditions for efficient transformation and regeneration and most of these studies employed *Agrobacterium*-mediated transformation procedure (**Table 3**). The general schematic protocol used for the *Agrobacterium*-mediated transformation of finger millet is presented in **Figure 4**. Only a limited number of studies were reported on the transformation of finger millet using a functionally active transgene. The details are discussed below.

**Table 3:** Details of various transformation studies reported in finger millet.

Name of the genotype	Promoter/ reporter gene used	Promoter/selectable marker	Functional gene used	Methods of transformation	Type of explants used	Application	References
PR202	*CaMV35S/ActI/UqI/RbcS/Ft GUS*	Nil	Nil	Biolistic	Leaf sheath segments	Testing the efficiency of various promoters in *GUS* expression	Gupta et al., 2001
PR202	*CaMV35S GUS*	*CaMV35S nptII*	Nil	*Agrobacterium*-mediated	Embryogenic seed	Establishment of transformation efficiency under different parameters	Sharma et al., 2011
^∗∗^	*CaMV35S GUS*	*CaMV35S bar*	*Antifungal protein (PIN)* gene of prawn	Biolistic	Shoot apex	Transgenics resistant to leaf blast disease	Latha et al., 2005
GPU45 and CO14	*CaMV35S GUS*	*CaMV35S hptII*	Nil	*Agrobacterium*-mediated	Shoot apex	Optimization of transformation using shoot apex	Ceasar and Ignacimuthu, 2011
GPU45 and CO14	*UqI GUS*	*UqI hptII*	Rice *chitinase* gene	*Agrobacterium*-mediated	Shoot apex	Transgenics resistant to leaf blast disease	Ignacimuthu and Ceasar, 2012
PR202	*CaMV35S GUS*	*CaMV35S hptII*	Nil	Biolistic	Green nodular calli	Optimization of biolistic mediated transformation protocol	Jagga-Chugh et al., 2012
Tropikanka and Yaroslav8	Nil	*CaMV35S bar*	*HvTUB1* and *TUAm1*	Biolistic and *Agrobacterium-*mediated	Embryogenic callus	Resistance to herbicides of the dinitroaniline family	Bayer et al., 2014
GPU28	*CaMV35S GUS*	*CaMV35S hptII*	Bacterial *mannitol-1-phosphate dehydrogenase* gene	*Agrobacterium*-mediated	Embryogenic callus	Tolerance to drought and salinity	Hema et al., 2014
GPU28	*CaMV35S GUS*	*CaMV35S hptII*	*PgNHX1, AVP1*	*Agrobacterium*-mediated	Embryogenic callus	Salinity tolerance	Jayasudha et al., 2014
CO(Ra)-14, PR-202, Try-1 and Paiyur2	*CaMV35S GUS*	*CaMV35S hptII*	Nil	*Agrobacterium*-mediated	Shoot apex	Optimization of transformation using direct plant regeneration	Satish et al., 2017

*Name of the marker and reporter genes with promoter, genotype, explant type and method of transformation used are given for each study. Any functional gene used is also indicated. AVP1, Arabidopsis vacuolar pyrophosphatase1; bar, phosphinothricin resistance; CaM35S, cauliflower mosaic virus 35s; Ft, promoter of C4 isoform of phosphoenolpyruate carboxylase gene from Flaveriatrinervia; gus or uidA, oxβ-glucuronidase; nos, nopaline synthase; hptII, hygromycin phosphotransferase; HvTUB1, Hordeum vulgare β1-tubulin; nptII, neomycin phosphotransferase; PgNHX1, Pennisetum glaucum Sodium hydrogen exchanger1; UqI, Ubiquitin promoter of maize); RbcS, ribulose-1,5-bisphosphate carboxylase small subunit gene promoter; TUAm1, α 1_tubulin mutant gene. ^∗∗^PGEC-2, IE-2576, IE-2367, IE-2366, IE-2683, IE-2684, IE-2851, IE-2861, IE-2333, IE-2995, IE-2300, IE-2675, IE-2340, IE-2983, IE-3242, IE-3020, IE-4673, IE-4683, IE-4120.*

### Genetic Improvement for Blast Resistance

A transgenic finger millet resistant to leaf blast disease was developed using *antifungal protein* (*PIN*) gene of prawn (Latha et al., 2005). The *PIN* gene was chemically synthesized and cloned into plasmid *pPin35S* under the control of *CaMV35S* promoter and transformed by biolistic method. Similarly, we have introduced a rice *Chitinase11* gene (*Chi11*) into genotype GPU45 of finger millet through *Agrobacterium*-mediated transformation to develop leaf blast resistance (Ignacimuthu and Ceasar, 2012). These two initial studies helped to develop the transgenic finger millet resistant to leaf blast disease. In both these reports, the transgenic plants overexpressing foreign gene exhibited resistance to leaf blast disease compared to non-transformed control plants. However, there are no reports available on transgenic finger millet resistant to neck and finger blasts. So screening of many other potential antifungal genes and gene pyramiding will be helpful to develop transgenic finger millet resistant to a wide spectrum of fungal diseases.

### Genetic Improvement for Abiotic Stress Tolerance

A salt-tolerant finger millet was developed using *sorghum vacuolar H^+^-pyrophosphatase* (*SbVPPase*) gene by *Agrobacterium*-mediated transformation. Overexpression of *SbVPPase* gene in finger millet enhanced the growth performance under salt stress. Jayasudha et al. (2014) also produced a transgenic finger millet by introducing *Na^+^/H^+^ antiporter* of *Pennisetum glaucum (PgNHX1)* and *Arabidopsis thaliana vacuolar H+-pyrophosphatase (AVP1)* for salinity stress tolerance through *Agrobacterium-*mediated transformation. The transgenic finger millet showed a higher level of salinity tolerance compared to wild type plants. *Porteresia coarctata*’s *serine-rich protein* (*PcSrp*) gene was overexpressed in finger millet under salinity condition (Mahalakshmi et al., 2006). The transgenic finger millet grown under 250 mM NaCl stress condition showed normal growth, flower and seed set rescuing from the saline stress (Mahalakshmi et al., 2006). Transgenic finger millet expressing a bacterial *mannitol-1-phosphate dehydrogenase* (*mtlD*) gene was developed through *Agrobacterium*-mediated transformation (Hema et al., 2014). Transgenic finger millet plants expressing *mtlD* gene had better growth under drought and salinity stress compared to wild-type. The transgenic plants also showed better osmotic stress tolerance with chlorophyll retention under drought stress compared to the wild-type plants (Hema et al., 2014).

It is evident that only a limited number of reports are available on overexpression of transgenes conferring tolerance to blast and other abiotic stresses in finger millet. More foreign genes need to be screened by overexpression for developing varieties resistant to multiple stresses. Most of these studies also focused on the introduction of foreign genes and phenotyping under a specific stress. High resolution studies, like subcellular localization of foreign gene and fusion of promoters of finger millet with reporter genes [GUS, green fluorescent protein (GFP), etc.] are yet to be performed in finger millet. The recently released WGS will be helpful to design such studies, especially those focusing on isolation of native promoters for functional analysis by fusing them with reporter genes. This will help to perform studies in line with those performed in model plants like rice and *A. thaliana* for functional validation of key genes and their promoters which will be useful to identify key genes and signals involved in grain filling of nutrients, drought tolerance, fungal resistance, etc.

## Molecular Marker-Assisted Breeding

Molecular markers are one of the important tools employed for the identification and improvement of particular traits. The DNA-based markers provide foundation for a wide range of molecular marker techniques, which are being widely used in the crop breeding program (Babu et al., 2007). Plant breeding backed by molecular markers helps to track traits more precisely when compared to conventional breeding. Several reports are available for the analysis of genetic diversity and QTL in finger millet using molecular markers which are discussed below.

### Genetic Diversity Analysis

The analysis of genetic diversity is crucial for crop improvement as it reveals the details of genetic relationships and provides insights for sampling of breeding populations (Mohammadi and Prasanna, 2003). Genetic diversity analysis helps to understand the relationships of genotypes around the world at genetic level and will aid in the selection of suitable genotypes for breeding programs (Babu et al., 2017). As finger millet is cultivated under diverse climatic conditions in Asia and Africa, analysis of genetic diversity helps to understand the genome variation between genotypes and subsequent population development for molecular marker analysis. The genotypes that are adapted to various biotic and abiotic stresses have more allele variation compared to susceptible genotypes. The genotypes having greater allele variation are being used for breeding programs. Randomly amplified polymorphic DNA (RAPD), restriction fragment length polymorphism (RFLP), and simple sequence repeats (SSR) markers were frequently used for the analysis of genetic diversity in finger millet (Parani et al., 2001; Fakrudin et al., 2004; Babu et al., 2014a) (Supplementary Table S2). Gupta et al. (2010) analyzed three genotypes of finger millet with variable seed coat color (brown, white, and golden) by studying morphological, physiological, and biochemical characteristics using 10 RAPD and 10 inter simple sequence repeats (ISSR) markers (Gupta et al., 2010). RAPD markers showed better polymorphism than ISSR markers. To investigate the genetic diversity of 32 finger millet genotypes, 45 RAPD primers were used (Patil and Kale, 2013). Out of 45 primers, 25 primers showed polymorphism and maximum genetic diversity was identified in VL149, KOPN 161, 338, and 929. The genetic diversity and population structure were assessed in 128 genotypes of finger millet collected from various geographical regions using 25 RAPD markers (Ramakrishnan et al., 2016b). Following this, genetic variation and population structure and relationship were evaluated between the Indian and non-Indian genotypes using 72 genomic SSR primers (Ramakrishnan et al., 2016a). Molecular variance and population structure in 42 genotypes of finger millet collected from different geographical regions of southern India were analyzed using 10 RAPD, 9 ISSR, and 36 SSR markers (Rajendran et al., 2016). These genotypes with diversity information can be used as parents of interest and may be crossed with elite material to develop new breeding population.

Although the PCR based markers were successfully used in the past for genetic diversity analysis, they have some limitations as the development of precise primers is very difficult (Arif et al., 2010). Alternatively, SNP based diversity analysis has been used in recent years for several plants for high throughput analysis of genetic diversity (Jeong et al., 2013; Ren et al., 2013; Tang et al., 2016). With no surprise, such studies have not yet been attempted in finger millet. Hopefully, the recently released WGS will aid in the development of SNP based diversity analysis in finger millet accessions (**Figure 3**). This will help to choose the genotypes for breeding and marker development based on SNP.

### Identification of QTLs for Agronomical Traits

**FIGURE 5:**
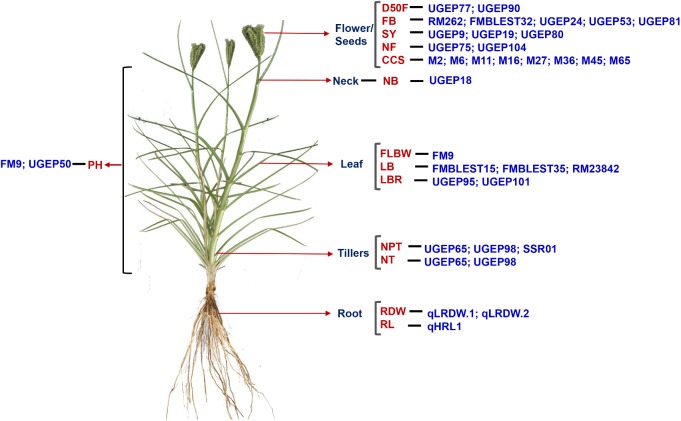
Identification of QTLs for important agronomical traits in finger millet. QTLs identified for various traits in different tissues are indicated with a pictorial representation. CCS, calcium content in seed; D50F, days to flowering (50%); FB, finger blast; FLBW, Flag leaf blade length; LB, leaf blast; LBR, leaf blast resistant; NB, neck blast; NF, number of fingers; NPT, number of productive tillers; NT, number of tillers; PH, plant height; RDW, root dry weight; RL, root length; SY, seed yield.

The microsatellite markers have been used to identify the agronomically important traits in finger millet such as grain yield, disease resistance, drought resistance and nutritional quality (**Figure 5**). Association mapping based identification of QTLs related to nutritional traits are thereby helpful in bio-fortification programs for ameliorating nutritional deficiencies (Kumar et al., 2015). For e.g., a total of 9 QTLs associated with Ca content were identified in 113 genotypes of finger millet using 23 anchored SSR markers (Kumar et al., 2015). Hence, determination of QTLs controlling these traits along with the candidate genes that cause deviation in Ca accumulation are essential for the successful incorporation into breeding and transgenic strategies. In another study, 46 genomic SSR markers were used to identify 4 agro-morphological traits such as basal tiller number, days to 50% flowering, flag leaf blade width, and plant height in 190 finger millet genotypes (Babu et al., 2014a) (**Figure 5**). The same group also identified four QTLs (UGEP81, UGEP24, FMBLEST32, and RM262) in the same genotypes of finger millet using 104 SSR markers (Babu et al., 2014c). In the same year, two QTLs (OM5 and FM8) were identified for tryptophan content and one QTL (FMO2EST1) for protein content in the aforementioned genotypes of finger millet using 120 SSR markers and these QTLs were linked to *opaque2 modifiers* (*Opm*) gene (Babu et al., 2014b). Tryptophan and lysine are the amino acids used in the biosynthesis of proteins. In cereal endosperm, these amino acids are deficient because it generally contains 1.5–2% lysine and 0.25–0.5% tryptophan, whereas 5% lysine and 1.1% tryptophan are required for optimal human nutrition. Finger millet contains high amount of tryptophan compared to other cereals. In view of this, identification of the QTLs linked to *Opm* gene responsible for the tryptophan content could be a major target for further improvement of the quality of finger millet germplasm. Further, 7 QTLs were found to be associated with seven agronomic traits, including productive tillers, seed yield, leaf blast resistance and number of tillers by 87 genomic SSR markers in 128 genotypes of finger millet (Ramakrishnan et al., 2016c). Recently, four QTLs (qLRDW.1, qLRDW.2, qHSDW.1, and qHRL.1) associated with root dry weight, shoot dry weight, and root length were identified in finger millet by association mapping under P deficient and P sufficient conditions (Ramakrishnan et al., 2017). In seedling stage, shoot and root growths were severely affected by P deficiency. Hence, the P deficiency tolerance in the seedling stage is an essential trait that needs to be used in finger millet cultivars (Ramakrishnan et al., 2017). This study provides input to breed low P-tolerant genotypes in finger millet using marker-assisted selection, and selected germplasm lines can be used either as cultivars for marginal lands where P deficiency is prominent or as donors for P starvation tolerance QTLs for breeding.

In finger millet, only the association mapping of populations was used so far for QTL studies. There is a crucial need to develop the linkage maps of finger millet for the identification of QTL, since it will play a major role in identifying the agronomically important traits. Moreover, the high throughput QTL mappings have not yet been attempted in finger millet due to lack of the WGS. Genome-wide association study (GWAS) has been emerging as a powerful tool in the identification of QTLs based on WGS. Several QTLs have been identified based on GWAS in cereals like rice (Huang et al., 2010, 2011, 2016; Zhang et al., 2018), barley (Visioni et al., 2013; Matthies et al., 2014; Fan et al., 2016; Bellucci et al., 2017) and maize (Mahuku et al., 2016; Wang et al., 2016). Development of low cost and high throughput genome sequencing technologies together with the availability of WGS will aid in the development of GWAS in finger millet in the coming years (**Figure 3**). This will be highly beneficial for dissecting QTLs and associated SNPs more precisely for key traits of finger millet including grain Ca content.

## Functional Characterization of Key Genes

Functional characterization of genes with key traits has been considered essential for developing varieties with improved traits. Only preliminary attempts have been made in finger millet for such studies (Supplementary Table S3). The recent developments in genomic research are expected to play a key role in the identification and characterization of candidate genes involved in nutrient signaling and transport in finger millet (Sood et al., 2016). As finger millet has 10-fold higher Ca in seeds compared to other cereals, dissection of key genes and signals involved in grain Ca filling will be important for nutrient enrichment of other cereals. The preliminary studies reporting the identification and expression analysis of candidate genes in finger millet are discussed below.

### Genes Involved in Ca Transport

Ca is a vital macronutrient for growth and development of plants as well as humans and animals. Ca is the third most important nutrient available in the soil and is required for normal growth of plants. In finger millet, maximum Ca is present in aleurone layer, followed by seed coat and embryo (Nath et al., 2013). Elevated level of Ca is also associated with higher expression of Ca-signaling transporter genes (Carter et al., 2004). Although there is no active transpiration stream within cells of the mature embryo, nutrient transfer between maternal and filial tissues is restricted to the apoplast (Patrick and Offler, 2001); therefore, changes in apoplastic Ca levels of the maternal plant could be reflected in the mature embryo or seed coat, which may be governed by Ca^2+^ transporter genes. So characterization of key genes involved in Ca accumulation will be helpful in transferring the same trait to other millets and non-millet cereals. Expression levels of key genes involved in Ca transport such as *Ca2^+^/H^+^ antiporter* (*CAX1*), *two pore channel1* (*TPC1*), *calmodulin (CaM)-stimulated type IIB Ca2+ ATPase* and *two CaM dependent protein kinase* (*CaMK1* and *CaMK2*) have been analyzed in 2 finger millet genotypes of contrasting Ca traits (GP-1, low Ca and GP-45, high Ca) (Mirza et al., 2014). The same group also identified 82 Ca sensor *genes* from the transcriptome of developing spikes of both genotypes GP-1 and GP-45 (Singh U.M. et al., 2014). As an outcome of this, the expression of 24 genes was higher in the pooled spike sample of genotype GP-45 while the expression of 11 genes was higher in the pooled spike sample of genotype GP-1. Twenty-four genes were highly expressed in the developing spikes of GP-45, seven encoded for *CaML*, two for *CRK*, five for *CBL*, seven for *CIPK*, and four for *CDPK* genes. Another report in the following year by the same group reported the characterization of *Ca^2+^ transporter* gene family in these two genotypes of finger millet. Whole genome and transcriptome profiling was also performed in the developing spikes of finger millet to find key genes involved in Ca*^2+^* transport (Singh U.M. et al., 2015). More recently, *CIPk24* gene was also characterized in these two genotypes of finger millet (Chinchole et al., 2017). This gene was overexpressed in root, shoot, leaf and developing spike tissues of GP-45 compared to GP1. Nine SNPs and one extra beta sheet domain as well as differences in vacuolar localization were identified through *in silico* analyses using the genomes of other model plants. Both EcCBL4 and EcCBL10 were found to show strong binding affinity with *EcCIPK24* (GP-1) compared to *EcCIPK24* (GP-45). It has been predicted that by activating EcCAX1b protein, EcCIPK24 can play an important role in high seed Ca accumulation (Chinchole et al., 2017).

Most of these studies were performed before the release of WGS of finger millet. So the complete list of genes involved in Ca sensing and transport has been analyzed in the primary article reporting the details of finger millet genome (Hittalmani et al., 2017). With no doubt, the complete genome sequence will aid in the functional characterization of key genes involved in Ca*^2+^* transport especially those mediate grain filling. High resolution studies involving reverse genetics approaches will help to dissect the complex mechanisms involved in Ca*^2+^* transport in finger millet. To this end, recently popularized tools like CRISPR/Cas9 may be helpful to develop mutants with defects in key genes of Ca transport and grain filling (**Figure 3**) since this technique demands WGS to avoid any off-target effects (Ceasar et al., 2016). CRISPR/Ca9 has been successfully applied in many plants for such studies.

### Genes Involved in N Metabolism

A few studies also reported the analysis of key genes involved in N transport in finger millet. Expression of *prolamin-binding factor DNA binding with one finger only* (*PBF Dof*) TF involved in regulation of seed protein storage was analyzed in different tissues like root, stem and flag leaf at vegetative stage and developing spikes of three finger millet genotypes (PRM-1, PRM-701, and PRM-801) with differing seed protein content and color (Gupta et al., 2011). The expression of this gene was relatively higher in developing spikes than in other tissues in all three genotypes. Interestingly, the grain protein content of these genotypes is directly related to higher expression of *PBF Dof* at early stages of growth (Gupta et al., 2011). Expression profile of key genes *Eleusine coracana high-affinity nitrate transporter* (*EcHNRT2*), *Ec low-affinity nitrate transporter* (*EcLNRT1*), *Ec nitrate reductase* (*EcNADH-NR*), *Ec glutamine synthetase (EcGS), Ec glutamine oxoglutarate aminotransferase* (*EcFd-GOGAT*) and *Ec DNA binding with one finger* 1 (*EcDof1*), involved in N uptake and assimilation were analyzed in two genotypes with contrasting (GE-1437, low-protein and GE-3885, high-protein) grain protein content (Gupta et al., 2013). Except *EcHNRT2*, remaining 5 genes were induced in the leaves of GE-3885 within 30 min of exposure to N deficiency. *EcNADH-NR* was found to be overexpressed in roots of GE3885 when the plans were exposed to increasing nitrate concentrations but not in GE-1437. This study revealed that GE-3885 might be a quick sensor of nitrogen compared to low-protein genotype (Gupta et al., 2013). In the following year, the same group also analyzed the expression pattern of *EcDof1* and *EcDof2* in the same genotypes (GE3885 and GE1437) (Gupta et al., 2014). *Dof1* and *Dof2* are TFs having opposite roles in regulation of genes related to C and N metabolism. The *EcDof1*/*EcDof2* ratio was higher in the roots of GE-3885 than in GE-3885 indicating higher activation of genes involved in N uptake and assimilation resulting in high grain protein accumulation (Gupta et al., 2014).

### Genes Involved in Carbon (C) Metabolism

Expression analysis was performed for some of the genes involved in C metabolism, such as c*hlorophyll a/b binding protein* (*Cab*), *Rubisco* (*RBCS*), *phosphoenol pyruvate carboxylase* (*PEPC*), *phosphoenol pyruvate carboxykinase* (*PEPC*-k), *malic enzyme* (*ME*), *sucrose phosphate synthase* (*SPS*), *pyruvatekinase* (*PK*), *pyruvate dikinase* (*PPDK*), *14-3-3* and *sensor protein kinase 1* (*SnRK1*) and co-expression of these genes with *Dof1*, in the same genotypes (GE-1437 and GE-3885) used in the above studies (Kanwal et al., 2014). Oscillations of expression of these genes were studied under light-dark conditions. The expression of these genes in both genotypes oscillated confirming their control by an endogenous clock. But the genes such as *Cab*, *RBCS* and *PPDK* showed no oscillations which might be due to induction by light. Expression of *Dof1* was higher in GE-3885 (higher grain protein genotype) along with other genes involved in C metabolism suggesting that *Dof1* regulates the expression of light inducible genes and controls the grain protein content in finger millet (Kanwal et al., 2014). This is the only report available on validation of genes involved in C metabolism. The WGS of finger millet will help to identify and characterize more genes in involved in C metabolism in near future.

### Genes Involved in Phosphate Transport

Four *phosphate transporter 1* (*EcPT1* to *EcPT4*) genes were identified and their expression was analyzed in three genotypes (RagiKorchara, Khairna, and VHC 3611) of finger millet (Pudake et al., 2017). The expression of these genes was validated under different regimes of inorganic phosphate (Pi) and under the colonization of *arbuscular mycorrhizae fungus* (AMF). It was found that *EcPT1* transcript levels were about fivefold higher in roots and leaves under deplete Pi than control. *EcPT3* gene was induced under phosphate stress in both leaves and roots. *EcPT4* genes was found to be induced by AMF in root tissues (Pudake et al., 2017). So far, only 4 *EcPT1* genes have been identified in finger millet. But each plant seems to possess more than 10 such genes (Baker et al., 2015). Even a close relative, foxtail millet has been reported to possess 12 PT genes which have been characterized for expression pattern, P transport assay in yeast and *in planta* function by downregulation through RNAi (Ceasar et al., 2014, 2017). These 4 PT genes were identified in finger millet based on the partial transcript sequences. Recently released WGS will be helpful for the genome-wide identification and functional characterization of all PT genes of finger millet.

### Genes Involved in Abiotic Stress Tolerance

Finger millet has been considered as a drought-hardy crop due to its adaptation for semi-arid tropical climate. Efforts have been made to characterize the key genes involved in drought tolerance and to utilize them for further applications. Drought stress is one of the most important abiotic factors affecting plant growth and productivity. Singh R.K. et al. (2015) made an effort to characterize the drought-responsive gene *EcDehydrin7* of finger millet by isolating and overexpressing it in tobacco. Tobacco plants overexpressing *EcDehydrin7* conferred tolerance to drought. Seven drought responsive genes (including *metallothionein*, *farnesylated protein ATFP6*, *protein phosphatase 2A*, *RISBZ4* and *farnesyl pyrophosphate synthase*) were found to be overexpressed in genotype GPU-28 under drought stress (Parvathi et al., 2013). These genes are believed to play crucial roles in drought tolerance and further characterization of these genes will help to identify any novel signals involved in drought tolerance in finger millet. A drought response regulatory gene of finger millet, *TBP Associated Factor6* (*EcTAF6*) was identified by screening cDNA library of finger millet and its expression in response to various stresses was analyzed in finger millet genotype GPU-28 (Parvathi and Nataraja, 2017). When the seedlings were exposed to NaCl, PEG and methyl viologen (oxidative stress), the normal growth was inhibited and *EcTAF6* was found to be significantly induced under these abiotic stresses when compared to the control (Parvathi and Nataraja, 2017). Drought responsive genes have also been identified and validated using drought responsive transcriptome by cDNA subtraction in finger millet (Ramegowda et al., 2017). One such potential gene, *EcGBF3* was characterized by ectopic expression in *A. thaliana*. Overexpression of *EcGBF3* in *A. thaliana* improved tolerance to osmotic, saline and drought stresses in *Atgbf3* mutant lines (Ramegowda et al., 2017). This study also indicated the difficulty in generating mutant lines in finger millet for such functional genomics studies; so it was analyzed using a model plant *A. thaliana*.

Several salinity stress responsive genes were identified in leaves of two contrasting finger millet genotypes viz., Co-12 (susceptible) and Trichy 1 (tolerant) under salinity condition through RNAseq (Rahman et al., 2014). The same group also reported that overexpression of *EcNAC67* TF in rice improved salinity and drought tolerances (Rahman et al., 2016). A stress responsive NAC gene (*EcNAC1*) was found to be highly up-regulated in response to salinity stress and was reported to be involved in tolerance against salinity and other abiotic stresses (Ramegowda et al., 2012). Two abiotic stress responsive TFs belonging to bZIP family (*EcbZIP60*) (Babitha et al., 2015a) and Basic helix-loop-helix (bHLH) family (*EcbHLH57*) (Babitha et al., 2015b) were identified in GPU-28 genotype of finger millet under drought, osmotic, salt and methyl viologen (MV) stresses. Nagarjuna et al. (2016) identified and characterized *CBL interacting protein kinase31* (*EcCIPK31-*like) gene responsible for drought tolerance in finger millet. A *TATA box Binding Protein* (TBP)-*Associated Factors* (TAFs) gene (*EcTAF6*) was identified in GPU-28 genotypes of finger millet under drought stress (Parvathi and Nataraja, 2017). More recently, a novel endoplasmic reticulum specific bZIP TF gene of finger millet (*EcbZIP17*) was isolated and overexpressed in tobacco (Ramakrishna et al., 2018). The tobacco plants overexpressing *EcbZIP17* exhibited tolerance to saline and heat stresses as compared to wild type plants.

These are the preliminary studies reported in finger millet on identification and validation of candidate genes. Unfortunately, these genes have not yet been characterized further in finger millet using reverse genetic tools as in model plants like rice and *A. thaliana*. It may be due to lack of WGS as one needs to design precise genomic targets for such studies. The recently released WGS is expected to help for such reverse genetic approaches like development of mutants using CRISPR, functional characterization by promoter reporter fusions, localization studies and heterologous expression in yeast mutants, etc. Overall, WGS of finger millet is expected to help to perform many high resolution studies to understand the function of genes involved in nutrient signaling and abiotic stress responses and could be tapped for breeding programs to develop improved finger millet.

## Conclusion and Future Prospects

Finger millet is a nutrient rich and drought hardy crop majorly cultivated and consumed by resource poor farmers in the developing countries of Asia and Africa. Only a limited number of genomic resources are available till date due to lack of WGS. Although finger millet has been considered as a climate resilient crop for the developing world, recent studies indicated that this crop is also vulnerable to drought, saline and low nutrient stresses in addition to fungal blast. Only a limited number of studies has been performed on characterization of functionally important genes of finger millet, before the release of WGS. WGS of two different finger millet genotypes were released recently (Hittalmani et al., 2017; Hatakeyama et al., 2018). This will help to design many high resoultion studies like those performed in other model plants such as rice and *A. thaliana* and WGS may change the course of finger millet research in future. The new genomic resource is expected to enrich the finger millet research in many spheres including dissection of key traits involved in nutrient enrichment and dorught tolerance using GWAS, genetic diversity analysis based on SNP, characterization of genes by reverse genetic studies using precise mutants using genome editing techniques like CRISPR/Cas9, accelerated functional gemomics studies such as promoter fusion of key genes with reporters like GFP for localication and spatial expression analysis, tissues specific transcriptome analysis to identify key regulatory genes of nutriant signaling and high thoroughput proteomics research to idenify the proteins associated with key agronomical functions. Overall, the recently released WGS of finger millet is expected to augment the finger millet research for its breeding and improvement. Many genes and proteins involved in the transport of key nutrients viz. Ca, P, N can be characterized in finger millet with the help of WGS. This will help to understand the key genes and regulatory networks involved in nutrient transport and can be harnessed for nutrient enrichment of other millets and non-millet cereals which will help to conserve nutrient security of growing world population.

## Author Contributions

SAC conceptualized the manuscript. TM, TPAK, and SAC wrote the manuscript. MR, LS, and GVR assisted, edited, and updated the manuscript. SI contributed critically in revising and improving the manuscript for publication.

## Conflict of Interest Statement

The authors declare that the research was conducted in the absence of any commercial or financial relationships that could be construed as a potential conflict of interest.

## Footnotes

1 https://phytozome.jgi.doe.gov/pz/portal.html
