# Supplementary material for: Finger Millet [Eleusine coracana (L.) Gaertn.] Improvement: Current Status and Future Interventions of Whole Genome Sequence
Source: Front Plant Sci. 2018 Jul 23;9:1054. doi: 10.3389/fpls.2018.01054 (PMC6064933; doi:10.3389/fpls.2018.01054)
Supplement: Supplementary file 1 [file Table_1.DOCX]

**Supplementary Table S1: Details of various *in vitro* studies reported in finger millet. Information on type of *in vitro* study, name of the genotype, type of explant, basal media and hormones used for each type of study are indicated with respective references.**

| **Methods of regeneration** | **Name of the genotype** | **Explants** | **Media composition**  **for callus induction** | **Percentage of callus induction** | **Media composition for shoot induction** | **Total number of shoots** | **Media composition for root induction** | **Total number of roots** | **References** |
| --- | --- | --- | --- | --- | --- | --- | --- | --- | --- |
| Indirect organogenesis | AKP 7 | LS | MS + 4 mg l^-1^ 2,4-D | x | MS + 0.25 mg l^-1^ 2,4-D | 33 | MS alone | x | Mohanty et al., 1985 |
|  |  | MC | MS + 2 mg l^-1^ 2,4-D | x |  | 29 |  |  |  |
|  | Dibysinha |  |  |  |  |  |  |  |  |
|  |  | LS | MS + 4 mg l^-1^ 2,4-D | x |  | 30 |  |  |  |
|  |  | MC | MS + 2 mg l^-1^ 2,4-D | x |  | 29 |  |  |  |
|  |  |  |  |  |  |  |  |  |  |
|  |  |  |  |  |  |  |  |  |  |
|  | CO-9, CO-12, C0-13 | MSD | MS + 4.0 mg l^-1^ picloram + 0.5 mg l^-1^ Kin | x | MS alone | x | NA | x | Pius et al., 1999 |
|  |  |  |  |  |  |  |  |  |  |
|  |  |  |  |  |  |  |  |  |  |
|  | PR - 202 | MSD | MS + 4.0 mg l^-1^ 2,4-D | 45.5 | MS alone | x | MS alone | x | Gupta et al., 2001 |
|  |  |  |  |  |  |  |  |  |  |
|  |  |  |  |  |  |  |  |  |  |
|  | PR-202 | MSD | MS + 2.0 mg l^-1^ 2,4-D + 0.5 mg l^-1^ Kin | 67 | x | x | x | x | Kumar et al., 2001 |
|  |  | ME |  | 63 | x | x | x | x |  |
|  |  | IMI | MS + 1.0 mg l^-1^ 2,4 D | 18 |  |  |  |  |  |
|  |  | IME | MS + 2.0 mg l^-1^ 2,4-D + 0.5 mg l^-1^ Kin | 74 | MS + 1.0 mg l^-1^ GA3 | 81.2 | MS + 1.0 mg l^-1^ GA3 | x |  |
|  | L-216 | MSD |  | 83 | x | x | x | x |  |
|  |  | ME |  | 82 | x | x | x | x |  |
|  |  | IMI | MS + 2.0 mg l^-1^2,4,5 - T + 0.2 mg l^-1^ BAP | 42 | x | x | x | x |  |
|  |  | IME | MS + 2.0 mg l^-1^ 2,4-D + 0.5 mg l^-1^ Kin | 93 | MS + 1.0 mg l^-1^ GA3 | 94.2 | MS + 1.0 mg l^-1^ GA3 | x |  |
|  | GE 4971 | MSD |  | x | x | x | x | x |  |
|  |  | ME |  |  | x | x | x | x |  |
|  |  | IMI | MS + 1 mg l^-1^ 2,4,5 - T + 0.2 mg l^-1^ BAP | 27 | x | x | x | x |  |
|  |  | IME | MS + 2 mg l^-1^ 2,4-D + 0.5 mg l^-1^ Kin | x | MS+ 1.0 mg l^-1^ GA3 | 67.8 | MS + 1.0 mg l^-1^ GA3 | x |  |
|  |  |  |  |  |  |  |  |  |  |
|  | ** | SA | MS + 2.0 mg l^-1^ 2,4-D + 0.25 mg l^-1^ kin | 93.0 – 98.8 | MS + 1.0 mg l^-1^ BAP + 1.0 mg l^-1^ Kin | x | MS + 1.0 mg l^-1^ BAP + 1.0 mg l^-1^ Kin | x | Latha et al., 2005 |
|  |  |  |  |  |  |  |  |  |  |
|  |  |  |  |  |  |  |  |  |  |
|  | PR-202 | ME | MS + 2, 4-D (9.0 µM) + Kin (2.3 µM). | x | MS + GA3  (2.9 µM) | 39 | MS + GA3  (2.9 µM) | x | Kothari Chajer et al., 2008 |
|  |  |  |  |  |  |  |  |  |  |
|  |  |  |  |  |  |  |  |  |  |
|  | CO-7 | SAM | MS + 4.0 mg l^-1^ 2,4-D + 0.5 mg l^-1^ Kin | 55.3 | MS + 1.0 mg l^-1^ TDZ  + 1.0 mg l^-1^ Kin | 16.4 | MS + 1.0 mg l^-1^ TDZ + 1.0 mg l^-1^ Kin | x | Ceasar and Ignacimuthu 2008 |
|  | CO-9 |  |  | 68.3 |  | 17.3 |  |  |  |
|  | CO-13 |  |  | 70.3 |  | 19.6 |  |  |  |
|  | CO-14 |  |  | 90.6 |  | 23.8 |  |  |  |
|  | GPU-26 |  |  | 86.9 |  | 21.3 |  |  |  |
|  | GPU-28 |  |  | 78.6 |  | 20.9 |  |  |  |
|  | GPU-45 |  |  | 98.6 |  | 26.5 |  |  |  |
|  | GPU-48 |  |  | 85.3 |  | 22.6 |  |  |  |
|  |  |  |  |  |  |  |  |  |  |
|  |  |  |  |  |  |  |  |  |  |
|  | IPGSM 10 | EC | MS + 2.0 mg l^-1^ 2,4-D | 93.33 | MS + 2.0 mg l^-1^ BAP | 4.2 | MS + 2 mg l^-1^, IBA + 0.1 mg l^-1^ BAP | x | Patil et al., 2009 |
|  | IPGSM 15 |  |  | 56.67 |  | 2.5 |  |  |  |
|  | IPGSM 17 |  |  | 55.00 |  | 3.4 |  |  |  |
|  | IPGSM 18 |  |  | 71.67 |  | 0.0 |  |  |  |
|  | IVT 4 |  |  | 73.33 |  | 3.5 |  |  |  |
|  | IVT 10 |  |  | 95.00 |  | 3.1 |  |  |  |
|  | IVT 16 |  |  | 58.33 |  | 3.7 |  |  |  |
|  | IVT 27 |  |  | 43.33 |  | 3.5 |  |  |  |
|  | GOA 8/1 |  |  | 43.33 |  | 0.0 |  |  |  |
|  | Dapoli 1 |  |  | 56.67 |  | 2.7 |  |  |  |
|  | WARC12/4 |  |  | 63.33 |  | 2.7 |  |  |  |
|  | AVTE 11 |  |  | 61.67 |  | 0.0 |  |  |  |
|  |  |  |  |  |  |  |  |  |  |
|  | PR - 202 |  | MS + 2.0 mg l^-1^ 2,4-D + 0.5 mg l^-1^ Kin | x | MS + GA3  (2.9 µM) | x | MS + GA3  (2.9 µM) | x | Sharma et al., 2011 |
|  |  |  |  |  |  |  |  |  |  |
|  |  |  |  |  |  |  |  |  |  |
|  | PR - 202 |  | MS + 2.0 mg l^-1^ 2,4-D + 0.5 mg l^-1^ Kin | x | MS + 1.0 mg l^-1^ GA3 | x | MS + 1.0 mg l^-1^ GA3 | x | Jagga Chugh et al., 2012 |
|  |  |  |  |  |  |  |  |  |  |
|  |  |  |  |  |  |  |  |  |  |
|  | Tropikanka and Yaroslav_8 | MSD | MS + 2.0 mg l^-1^ 2,4-D + 0.5 mg l^-1^ Kin | x | MS alone | x | x | x | Bayer et al., 2014 |
|  |  |  |  |  |  |  |  |  |  |
|  |  |  |  |  |  |  |  |  |  |
|  | GPU28 | MSD | MS + 3.0 mg l^-1^ 2, 4-D + 0.5 mg l^-1^ BAP | x | MS + 0.5 mg l^-1^ BAP | x | MS + 0.5 mg l^-1^  BA + 0.1 mg l^-1^  NAA | x | Hema et al., 2014 |
|  |  |  |  |  |  |  |  |  |  |
|  |  |  |  |  |  |  |  |  |  |
|  | GPU28 | MSD | MS + 2.5 mg l^-1^ 2,4-D + 0.5 mg l^-1^ BAP | x | MS + 80 mM NH_4_ NO_3_ | x | ½ MS alone | x | Jayasudha et al., 2014 |
|  |  |  |  |  |  |  |  |  |  |
|  |  |  |  |  |  |  |  |  |  |
|  | PES 400 |  | MS + 1.5 mg l^-1^ 2, 4-D + 1.5 mg l^-1^ BAP. | 93 | MS + 3.0 mg l^-1^ of BAP | 27 | MS alone | x | Pande et al., 2015 |
|  |  |  |  |  |  |  |  |  |  |
|  |  |  |  |  |  |  |  |  |  |
|  | PES 400 | MSD | MS + 2.0 mg l^-1^ 2,4-D + 0.5 mg l^-1^ Kin | 95.6 | MS + 1.0 mg l^-1^ Kin + 0.5 mg l^-1^ GA3 | 8 | MS + 0.5 mg l^-1^ IBA + 0.5 mg l^-1^ NAA | 12.8 | Dosad and Chawla 2015 |
|  |  |  |  |  |  |  |  |  |  |
|  |  |  |  |  |  |  |  |  |  |
|  | Co 15 | MSD | MS + 5 mg l^-1^ 2, 4-D + 0.5 mg l^-1^ Kin. | 62.02 | MS + 1 mg l^-1^ GA3 | 9.2 | MS + 1 mg l^-1^ GA3 | 8.2 | Anju et al., 2016 |
|  | Co(Ra)14 |  | MS + 2 mg l^-1^ 2,4-D + 0.5 mg l^-1^ BAP | 64.93 |  | 7.2 |  | 7.1 |  |
|  | GPU 45 |  | MS + 2.5 mg l^-1^ 2, 4-D + 0.5 mg l^-1^ BAP | 55.73 |  | 8.8 |  | 6.5 |  |
|  | K252 |  |  | 48.13 |  | 6.5 |  | 6.8 |  |
|  |  |  |  |  |  |  |  |  |  |
|  |  |  |  |  |  |  |  |  |  |
| Somatic embryogenesis | CO(Ra)-14 | SAM | MS + 50% *Gracilaria edulis* | 91.5 | MS + 50% *Gracilaria edulis* | x | ½ MS + 60% *Gracilaria edulis* | x | Satish et al., 2016a |
|  | Hosur-1 |  |  | 78.2 |  |  |  |  |  |
|  | PR-202 |  |  | 96.3 |  |  |  |  |  |
|  | Try-1 |  |  | 84.1 |  |  |  |  |  |
|  |  |  |  |  |  |  |  |  |  |
|  |  |  |  |  |  |  |  |  |  |
|  | CO(Ra)-14 | ME | MS + 4.0 mg l^-1^ 2,4-D + 0.5 mg l^-1^ Kin | 95.6 | MS + 4.0 BAP + 0.2 mg l^-1^ 2, 4-D | 25.8 | ½ MS alone | x | Satish et al., 2016b |
|  |  | SAM |  | 71.7 |  | 23.8 |  |  |  |
|  | Paiyur-2 | ME |  | 54.2 |  | 15.6 |  |  |  |
|  |  | SAM |  | 60.0 |  | 18.9 |  |  |  |
|  | Try-1 | ME |  | 81.3 |  | 17.3 |  |  |  |
|  |  | SAM |  | 66.1 |  | 21.9 |  |  |  |
|  | GPU-25 | ME |  | 58.4 |  | 19.3 |  |  |  |
|  |  | SAM |  | 46.8 |  | 11.7 |  |  |  |
|  |  |  |  |  |  |  |  |  |  |
|  |  |  |  |  |  |  |  |  |  |
| Direct organogenesis | CO(Ra)-14 | SAM | x | x | MS + 17.6 µM BAP | 6.2 | 1/2 MS + 2.8 µM IAA | 5.4 | Satish et al., 2015 |
|  | Paiyur-2 |  |  |  |  | 4.5 | x | x |  |
|  | Try-1 |  |  |  |  | 4.9 | x | x |  |
|  |  |  |  |  |  |  |  |  |  |
|  |  |  |  |  |  |  |  |  |  |
|  | CO 9 | SAM | x | x | MS + 3.0 mg l^-1^ BAP | 26 | MS + 0.25 mg l^-1^ IAA | 7.63 | Babu et al., 2018 |
|  | CO (Ra) 14 |  |  |  |  | 22 |  | 4.31 |  |
|  | GPU 28 |  |  |  |  | 20 |  | 5.49 |  |

**^Abbreviations^** ^used< BAP, Benaylaminopurine; EC, epicotyl; GA3-giberrlic acid; IAA, indole-3-acetic acid, IBA, indole-3-butyric acid; IME, immature embryo; IMIs, immature inflorescences; Kin, kinetin; LS, leaf segments; MC, Mesocotyl; ME, mature embryos; MS, Murashige and Skoog; MSD, mature seeds; NA, Not applicable; NAA, naphthaleneacetic acid; SA, shoot apex; SAM, shoot apical meristem; TDZ, thidiozuron; 2,4-D , 2,4-dichlorophenoxyacetic acid; X, data not provided.^

**^PGEC-2, IE-2576, IE-2367, IE-2366, IE-2683, IE-2684, IE-2851, IE-2861, IE-2333, IE-2995, IE-2300, IE-2675, IE-2340, IE-2983, IE-3242, IE-3020, IE-4673, IE-4683, IE-4120.^

**References**

Anju, C., Rabindran, R., Velazhahan, R., and Ravikesavan, R. (2016). Callusing and regeneration in finger millet (*Eleusine coracana* (L.) Gaertn.) *Res. J. Agric. Sci.* 7, 324-329.

Jayasudha, B. G., Sushma, A. M., Prashantkumar, H. S., and Sashidhar, V. R. (2014). An efficient invitro agrobacterium mediated transformation protocol for raising salinity tolerant transgenic finger millet (*Eleusine coracana* (L.) Gaertn) *Plant Arch.* 14, 823-829.

Babu, G. A., Vinoth, A., and Ravindhran, R. (2018). Direct shoot regeneration and genetic fidelity analysis in finger millet using ISSR markers. *Plant Cell. Tiss. Organ Cult.* 132, 157-164. doi: 10.1007/s11240-017-1319-z

Bayer, G. Y., Yemets, A., and Blume, Y. B. (2014). Obtaining the transgenic lines of finger millet Eleusine coracana (L.). with dinitroaniline resistance. *Cytol. Genet.* 48, 139-144. doi: 10.3103/S0095452714030025

Ceasar, S. A., and Ignacimuthu, S. (2008). Efficient somatic embryogenesis and plant regeneration from shoot apex explants of different Indian genotypes of finger millet (*Eleusine coracana* (L.) Gaertn.). *In Vitro Cell. Dev. Biol.- Plant.* 44, 427-435. doi: 10.1007/s11627-008-9153-y

Dosad, S., and Chawla, H. S. (2015). In vitro plant regeneration from mature seeds of finger millet (*Eleusine coracana*) through somatic embryogenesis. *Ind J Plant Physiol.* 20, 360-367. doi: 10.1007/s40502-015-0191-2

Gupta, P., Raghuvanshi, S., and Tyagi, A. K. (2001). Assessment of the efficiency of various gene promoters via biolistics in leaf and regenerating seed callus of millets, *Eleusine coracana* and *Echinochloa crusgalli*. *Plant Biotechnol.* 18, 275-282.

Hema, R., Vemanna, R. S., Sreeramulu, S., Reddy, C. P., Senthil Kumar, M., and Udayakumar, M. (2014). Stable expression of mtlD gene imparts multiple stress tolerance in finger millet. *PLoS ONE* 9:e99110. doi: 10.1371/journal.pone.0099110

Jagga Chugh, S., Kachhwaha, S., Sharma, M., Kothari Chajer, A., and Kothari, S. (2012). Optimization of factors influencing microprojectile bombardment-mediated genetic transformation of seed-derived callus and regeneration of transgenic plants in *Eleusine coracana* (L.) Gaertn. *Plant Cell. Tiss. Organ Cult*. 109, 401-410. doi: 10.1007/s11240-011-0104-7

Kothari Chajer, A., Sharma, M., Kachhwaha, S., and Kothari, S. (2008). Micronutrient optimization results into highly improved in vitro plant regeneration in kodo (Paspalum scrobiculatum L.) and finger (*Eleusine coracana* (L.) Gaertn.) millets. *Plant Cell. Tiss. Organ Cult*. 94, 105-112. doi: 10.1007/s11240-008-9392-y

Kumar, S., Agarwal, K., and Kothari, S. (2001). In vitro induction and enlargement of apical domes and formation of multiple shoots in finger millet, *Eleusine coracana* (L.) Gaertn and crowfoot grass, *Eleusine indica* (L.) Gaertn. *Curr. Sci.* 81, 1482-1485.

Latha, A. M., Rao, K. V., and Reddy, V. D. (2005). Production of transgenic plants resistant to leaf blast disease in finger millet (*Eleusine coracana* (L.) Gaertn.). *Plant Sci.* 169, 657-667. doi: 10.1016/j.plantsci.2005.05.009

Mohanty, B., Gupta, S. D., and Ghosh, P. (1985). Callus initiation and plant regeneration in ragi (*Eleusine coracana* Gaertn.). *Plant Cell. Tiss. Organ Cult*. 5, 147-150.

Pande, A., Dosad, S., Chawla, H., and Arora, S. (2015). In-vitro organogenesis and plant regeneration from seed-derived callus cultures of finger millet (*Eleusine coracana*). *Braz. J. Bot.* 38, 19-23. doi: 10.1007/s40415-014-0102-1

Patil, S., Sawardekar, S., Bhave, S., Sawant, S., Jambhale, N., and Gokhale, N. (2009). Development of somaclones and their genetic diversity analysis through RAPD in Finger millet (*Eleusine coracana* L. Gaertn.). *Ind J. Genet.* *Plant Breed.* 69, 132-139.

Pius, J., Eapen, S., George, L., Rao, P., and Raut, R. (1999). Performance of plants regenerated through somatic embryogenesis in finger millet (*Eleusine coracana* Gaertn.). *Trop. Agric. Res. Exten.* 2, 87-90.

Satish, L., Ceasar, S. A., Shilpha, J., Rency, A. S., Rathinapriya, P., and Ramesh, M. (2015). Direct plant regeneration from in vitro-derived shoot apical meristems of finger millet (*Eleusine coracana* (L.) Gaertn.). *In Vitro Cell. Dev. Biol.-Plant.* 51, 192-200. doi: 10.1007/s11627-015-9672-2

Satish, L., Rathinapriya, P., Rency, A. S., Ceasar, S. A., Pandian, S., Rameshkumar, R., and Ramesh, M. (2016a). Somatic embryogenesis and regeneration using *Gracilaria edulis* and *Padina boergesenii* seaweed liquid extracts and genetic fidelity in finger millet (*Eleusine coracana*). *J Appl. Phycol.* 28, 2083-2098. doi: 10.1007/s10811-015-0696-0

Satish, L., Rency, A. S., Rathinapriya, P., Ceasar, S. A., Pandian, S., Rameshkumar, R., Rao, T. B., Balachandran, S., and Ramesh, M. (2016b). Influence of plant growth regulators and spermidine on somatic embryogenesis and plant regeneration in four Indian genotypes of finger millet (*Eleusine coracana* (L.) Gaertn). *Plant Cell. Tiss. Organ Cult*. 124, 15-31. doi: 10.1007/s11240-015-0870-8

Sharma, M., Kothari Chajer, A., Jagga Chugh, S., and Kothari, S. (2011). Factors influencing Agrobacterium tumefaciens-mediated genetic transformation of *Eleusine coracana* (L.) Gaertn. *Plant Cell. Tiss. Organ Cult*. 105, 93-104. doi: 10.1007/s11240-010-9846-x
