# Supplementary material for: Finger Millet [Eleusine coracana (L.) Gaertn.] Improvement: Current Status and Future Interventions of Whole Genome Sequence
Source: Front Plant Sci. 2018 Jul 23;9:1054. doi: 10.3389/fpls.2018.01054 (PMC6064933; doi:10.3389/fpls.2018.01054)
Supplement: Supplementary file 2 [file Table_2.DOCX]

**Supplementary Table S2.** Details on genetic diversity studies performed in various genotypes of finger millet using different molecular markers

| **No. of genotypes** | **Origin** | **Name of marker** | **No. of marker** | **No. of cluster** | **Genetic variation** | **No. of Sub-Population** | **References** |
| --- | --- | --- | --- | --- | --- | --- | --- |
| 26 | Africa , India | Mt DNA, RFLP | 1 ^Mt^ ; 5 ^RE^ | 3 ^A^ | - | - | Muza et al., 1995 |
| 12 | Africa , India | RAPD | 37 | 2 ^A^ | 0.55 ^B^ | - | Fakrudin et al., 2004 |
| 32 | India | RAPD | 50 | 2 ^A^ | 0.315-0.888 ^C^ | - | Babu et al., 2007 |
| 30 | India | RAPD | 13 | 2 ^A^ | 0.13-0.91 ^C^ | - | Das et al., 2007 |
| 15 | India | RAPD | - | - | - | - | Das et al., 2009 |
| 64 | Ethiopia , Eritrea | RAPD | 15 | 9 ^A^ | 0.585-0.984 ^C^; 0.83 ^B^ | - | Bezaweletaw 2011 |
| 32 | India | RAPD | 45 | - | - | - | Karad et al., 2013 |
| 128 | Asia, Europe, USA , Africa | RAPD | 25 | 4 ^A^ ; 12 ^D^ | 0.0085-0.81 ^C^ | 4 | Ramakrishnan et al., 2015 |
| 3 | India | RAPD , ISSR | 20 | - | - | - | Gupta R et al., 2010 |
| 83 | India, Malawi, Uganda , Kenya | RAPD, SSR , cytP(450) | 18^R^, 10^S^ and 10^P^ | - | 0.675^C^; 0.583^C^ and 0.631^C^ | - | Panwar et al., 2010b |
| 52 | India | RAPD, SSR , cytP(450) | 18^R^, 10^S^ and 10^P^ | - | 0.505^C^; 0.504^C^ and 0.499^C^ | - | Panwar et al., 2010a |
| 22 | USA, Asia , Africa | RFLP, RAPD , ISSR | 18^R^, 6^S^ and 6^I^ | - | - | - | Salimath et al., 1995 |
| 2 | - | Ribosomal DNA |  | - | - | - | Hilu 1995 |
| 79 | Uganda, Kenya , Asia , Africa | SSR | 45 | different | - | - | Dida et al., 2008 |
| 67 | Africa , India | SSR | 17 | 3 ^A^ ; 4 ^D^ | 0.471 ^B^ | 5 | Arya et al., 2013 |
| 103 | Africa , India | SSR |  |  |  |  | Nirgude et al., 2014 |
| 190 | Asia, Europe, USA , Africa | SSR | 74 | 3 ^A^ | 0.208-0.752 ^C^ | 4 | Babu et al., 2014 |
| 128 | Asia, Europe, USA , Africa | SSR | 87 | 3 ^A^ | 0.011-0.836 ^C^ | 3 | Ramakrishnan et al., 2016 |
| 52 | India | RAPD, SSR | 21 ^R^ ; 24 ^S^ | different | 0.819 ^B, R^  0.774 ^B, S^ | - | Kumar et al., 2012 |
| 24 | India | SSR | 35 | - | - | - | Nethra et al., 2014 |
| 48 | Asia , Africa | SSR | 70 |  | 0.7638 |  | Nnaemeka Obidiegwu 2014 |
| 28 | India, Africa, Uganda | EST-SSR | 21 | 2 ^A^ | - | - | Joshi et al., 2018 |
| 42 | India | RAPD, ISSR, SSR | 10, 15, 14 | 3, 4, 3 | 0.016, 0.01, 0.013 | - | Rajendran et al., 2016 |
| 32 | - | RAPD | 45 | 3 | 0.80 | 5 | Patil and Kale 2013 |
| 40 | - | ISSR, SSR | 30, 10 | 5 ,13 | - | - | Prabhu 2013 |
|  |  |  |  |  |  |  |  |

**^Abbreviation:^** ^A, major cluster; B, average gene diversity; C, range gene diversity or genetic similarity; D, sub-cluster; ISSR, inter simple sequence repeats; I, ISSR markers; cytP450, pairs of cytochrome P (450) gene based markers; RAPD, random amplified polymorphic DNA, R, RAPD; SSR, simple sequence repeats; S, SSR; Mt, mitochondrial; RE, restriction endonuclease.^

**References**

Arya, L., Verma, M., Gupta, V. K., and Seetharam, A. (2013). Use of genomic and genic SSR markers for assessing genetic diversity and population structure in Indian and African finger millet (*Eleusine* *coracana* (L.) Gaertn.) germplasm. *Plant Syst. Evol.* 299, 1395-1401. doi:10.1007/s00606-013-0822-x

Babu, B. K., Agrawalm P. K., Pandey, D., and Kumar, A. (2014). Comparative genomics and association mapping approaches for opaque2 modifier genes in finger millet accessions using genic, genomic and candidate gene-based simple sequence repeat markers. *Mol. Breed.* 34, 1261-1279. doi:10.1007/s11032-014-0115-2

Babu, B. K., Senthil, N., Gomezm S. M., Biji, K. R., Rajendraprasad, N. S., Kumar, S. S., and Babu, R. C. (2007). Assessment of genetic diversity among finger millet (*Eleusine coracana* (L.) Gaertn.) accessions using molecular markers. *Genet. Resour. Crop Evol.* 54, 399-404. doi:10.1007/s10722-006-0002-8

Bezaweletaw, k. (2011). Genetic diversity of finger millet [*Eleusine coracana* (L.) Gaertn] landraces characterized by random amplified polymorphic DNA analysis. *Innov. Syst. Des. Eng.* 2, 207-218

Das, S., Misra, R. C., Routi, G. R., Pattanaik, M. C., and Aparajita, S. (2009). Relationship of status of polymorphic RAPD bands with genotypic adaptation in early finger millet genotypes. *Afr. Crop Sci. J.* 17, 61-69

Das, S., Mishra, R. C., Rout, G. R., and Aparajita, S. (2007). Genetic variability and relationships among thirty genotypes of finger millet (*Eleusine coracana* L. Gaertn.) using RAPD markers. Zeitschrift fur Naturforschung C, *J. Biosci.* 62, 116-122. DOI:10.1515/znc-2007-1-220

Dida, M. M., Wanyera, N., Harrison, Dunn, M. L., Bennetzen, J. L., and Devos, K. M. (2008). Population structure and diversity in finger millet (*Eleusine coracana*) germplasm. *Trop. Plant Biol.* 1, 131-141. doi:10.1007/s12042-008-9012-3

Fakrudin, B., Shashidhar, H., Kulkarni, R., and Hittalmani, S. (2004). Genetic diversity assessment of finger millet, *Eleusine coracana* (Gaertn.), germplasm through RAPD analysis. *PGR Newslett.* 138, 50-54.

Gupta, R., Verma, K., Joshi, D. C., Yadav, D., and Singh, M. (2010). Assessment of genetic relatedness among three varieties of finger millet with variable seed coat color using RAPD and ISSR markers. *Genet. Eng. Biotechnol. J*. 2, 1-9.

Hilu, K. W. (1995). Evolution of finger millet: evidence from random amplified polymorphic DNA. *Genome*. 38, 232-238. doi:10.1139/g95-028

Joshi, C., Subramanya, S., and Ravikumar, R. (2018). Evaluation of morphological and molecular diversity among the genotypes of *Eleusine africana*, cultivated and weedy types of finger millet (*Eleusine coracana* L. Gaertn.). *Int. J. Curr. Microbiol. App. Sci*. 7, 1836-1848.

Karad, S., Patil, J. V., and Kale, A. A. (2013). Study of genetic diversity in finger millet (*Elesuine Coracana* L.) genotypes using RAPD markers. *Int. J. Int. Sci. Inn. Tech. Sec. A*. 2, 31-36.

Kumar, A., Sharma, N., Panwar, P., and Gupta, A. (2012). Use of SSR, RAPD markers and protein profiles based analysis to differentiate *Eleusine coracana* genotypes differing in their protein content. *Mol. Biol. Rep.* 39, 4949-4960. doi:10.1007/s11033-011-1291-3

Muza, F. R., Lee, D. J., Andrews, D. J., and Gupta, S. C. (1995). Mitochondrial DNA variation in finger millet (*Eleusine coracana* L. Gaertn). *Euphytica*. 81, 199-205. doi:10.1007/BF00025434

Nethra, N., Gowda, R., Rajendra, P. S., Hittalmani, S., Ramanjini, G. P., and Chennakeshava, B. (2014). Utilization of SSRs to estimate the degree of genetic relationships in finger millet (*Eleusine coracana* L. Gaertn.) genotypes and subspecies. *SABRAO J. Breed. Genet*. 46, 136-149.

Nirgude, M., Babu, B. K., Shambhavi, Y., Singh, U. M., Upadhyaya, H. D., and Kumar, A. (2014). Development and molecular characterization of genic molecular markers for grain protein and calcium content in finger millet (*Eleusine coracana* (L.) Gaertn.). *Mol. Biol. Rep*. 41, 1189-1200. doi:10.1007/s11033-013-2825-7

Nnaemeka, Obidiegwu. O. (2014). Development and genotyping potentials of EST-SSRs in finger millet (*E.* *coracana* (L.) Gaertn.). *Int. J. Genet. Genomics* 2, 42-46. doi:10.11648/j.ijgg.20140203.12

Panwar, P., Nath, M., Yadav, V. K., and Kumar, A . (2010a). Comparative evaluation of genetic diversity using RAPD, SSR and cytochrome P450 gene based markers with respect to calcium content in finger millet (*Eleusine coracana* L. Gaertn.). *J. Genet*. 89, 121-133.

Panwar, P., Saini, R. K, Sharma, N., Yadav, D., and Kumar, A. (2010b). Efficiency of RAPD, SSR and cytochrome P450 gene based markers in accessing genetic variability amongst finger millet (*Eleusine coracana*) accessions. *Mol. Biol. Rep*. 37, 4075-4082. doi:10.1007/s11033-010-0067-5

Patil, J., and Kale, A. (2013) Study of genetic diversity in finger millet (*Elesuine coracana* L.) genotypes using RAPD markers. *Int. J. Int. Sci. Inn. Tech. Sec. A*. 2, 31-36.

Prabhu, R. (2013). Genetic diversity studies in Ragi [*Eleusine coracana* (L.) Gaertn.] with SSR and ISSR mrkers. *Mol Plant Breed.* 4, 141-145.

Rajendran, H. A. D., Muthusamy, R., Stanislaus, A. C., Krishnaraj, T., Kuppusamy, S., Ignacimuthu, S., and Al-Dhabi, N. A., (2016). Analysis of molecular variance and population structure in southern Indian finger millet genotypes using three different molecular markers. *J. Crop Sci. Biotech.* 19, 275-283. doi: 10.1007/s12892-016-0015-6

Ramakrishnan, M., Ceasar, S., Duraipandiyan, V., Al-Dhabi, N., and Ignacimuthu, S. (2016). Assessment of genetic diversity, population structure and relationships in Indian and non-Indian genotypes of finger millet (*Eleusine coracana* (L.) Gaertn) using genomic SSR markers. *SpringerPlus*. 5, 1-11. doi:DOI 10.1186/s40064-015-1626-y

Ramakrishnan, M., Ceasar, S. A., Duraipandiyan, V., Al-Dhabi, N. A., and Ignacimuthu, S. (2015). Using molecular markers to assess the genetic diversity and population structure of finger millet (*Eleusine coracana* (L.) Gaertn.) from various geographical regions. *Genet. Resour. Crop Evol.* 63, 361-376. doi:10.1007/s10722-015-0255-1

Salimath, S. S., de Oliveira, A. C., Godwin, I. D., and Bennetzen, J. L. (1995). Assessment of genome origins and genetic diversity in the genus *Eleusine* with DNA markers. *Genome*. 38, 757-763.
