# Supplementary material for: Finger Millet [Eleusine coracana (L.) Gaertn.] Improvement: Current Status and Future Interventions of Whole Genome Sequence
Source: Front Plant Sci. 2018 Jul 23;9:1054. doi: 10.3389/fpls.2018.01054 (PMC6064933; doi:10.3389/fpls.2018.01054)
Supplement: Supplementary file 3 [file Table_3.DOCX]

**Supplementary Table S3.** Details on identification and expression analysis of functionally important genes in finger millet for various functions

| **Gene/**  **Transcript Name** | **Function** | **Name & property of genotypes** | **Expression pattern, name of the genotype and tissue specificity** | **Mode**  **of analysis** | **References** |
| --- | --- | --- | --- | --- | --- |
|  |  |  |  |  |  |
| *PBF Dof* | Seed storage, protein content and color | HPC white: PRM-801,  MPC golden: PRM 701,  LPC brown: PRM-1 | **VS**  ↑ *PBF Dof*: roots of PRM-801, PRM-701and PRM-1 at 21 DAS  ↓ *PBF Dof*: stems of PRM-801 and leaves of PRM-701 & PRM-1 at 21 DAS  **DS**  ↑ *PBF Dof*: S2, S3 and S4 of PRM-801, PRM-701and PRM-1  ↓ *PBF Dof*: PRM-801(S3) PRM-701(S1) & PRM-1(S2) | RT-PCR, qRT-PCR, RNAseq | Gupta et al., 2011 |
|  |  |  |  |  |  |
| *EcNAC1* | Abiotic stress tolerance | GPU-28 | **VS**  ↑ leaves at 60% drought FC and 200 mM NaCl at 12 h  ↓leaves at 35% drought FC, 100 mM NaCl at 12 h and 24 h and 200 mM NaCl at 24h. | RT-PCR  qRT-PCR | Ramegowda et al. 2012 |
|  |  |  |  |  |  |
| *EcHNRT2,*  *EcNR,*  *EcGS,*  *EcFd-GOGAT* | Nitrogen  uptake and assimilation | HPG: GE-3885,  LPG: GE-1437 | **VS**  ↑*EcHNRT2, EcFd-GOGAT*: roots and shoots of GE-3885 and GE-1437 at 21 **DAS**  ↑*EcNR*: shoots of GE-3885 and GE- 1437 at 21 DAS  ↓*EcNR* and *EcGS*: roots of GE-3885 and GE-1437 at 21 DAS | RT-PCR,  qRT-PCR  RNAseq | Gupta et al., 2013 |
|  |  |  |  |  |  |
| *EcDof1, EcDof2* | Carbon and nitrogen metabolism | HPG: GE-3885,  LPG: GE-1437 | **VS**  **Roots**  ↑*EcDof1*: GE-1437 in 0.15 µM N and GE-3885 in 150 µM N conc.  ↓ *EcDof1*: GE-3885 and GE-1437 in 1.5 µM N conc.  ↑*EcDof2*: GE-1437 and GE-3885 in 1500 µM N conc.  ↓*EcDof2*: GE-1437 and GE-3885 in 0.15 µM N conc.  **Shoots**  ↑*EcDof1*: GE-3885 and GE-1437 under 15 µM N conc.  ↑*EcDof2*: GE-1437 in 1.5 µM N conc.  ↓ *EcDof2*: GE-3885 in 1.5 µM N conc.  ↑*EcDof1*: GE-1437 under dark & illumination and GE-3885 only in illumination  ↓*EcDof1*: GE-3885 under dark (19^th^ day 6h).  ↑*EcDof2*: GE-1437 in diurnal and GE-3885 in illumination  ↓*EcDof2*: GE-3885 and GE-1437 in dark | qRT-PCR, RNAseq | Gupta et al., 2014 |
|  |  |  |  |  |  |
| *Cab, RBCS PEPC, PPDK, PEPC-k, ME, SPS, PK,*  *14-3-3, SnRK1*  *Tubulin*, *Dof1* | Carbon metabolism | HPG: GE-3885,  LPG: GE-1437 | **DS**  ↑ All genes at S1-S4 stages in GE-3885  ↓ All genes at S1-S4 stages in GE-1437 | qRT-PCR  RNAseq | Kanwal et al., 2014 |
|  |  |  |  |  |  |
| *CAX1, TPC1, Ca2+ ATPase, CaMK1, CaMK2 CAM*,  *14-3-3* | Calcium uptake and translocation | HCaG: GP-45,  LCaG: GP-1 | **VS**  **Roots**:  ↑*CAX1, TPC1, Ca2+ATPase, CaMK1, CaMK2* in GP-45 at 90 DAS  ↑*CAX1, TPC1, Ca2+ATPase* in GP-1 at 60 DAS  ↓*CAM* and *14-3-3* in GP-1 and GP-45 at 30 DAS  **Leaves**:  ↑ *CAX1* and *14-3-3* in GP-45 at 60 DAS  **DS**  ↑*CAMK2*, *TPC1*, *Ca2^+^ATPase 14-3-3*, *CAX1* and *CAX3* in GP-45 at S1-S4 stages | qRT-PCR  RNAseq | Mirza et al., 2014 |
|  |  |  |  |  |  |
| *EcCaM1, EcCaML4,*  *EcCaML11,*  *EcCaML14,*  *EcCIPK9,*  *EcCIPK11,*  *EcCDPK14,*  *EcCDPK3* | Calcium sensor | HCaG: GP-45,  LCaG: GP-1 | **DS**  ↑*EcCaM1, EcCaML4, EcCaML14* and *EcCDPK14* in GP-45 at S2 and S4 stage.  ↑*EcCDPK3* of GP-1 at S1 to S4 stages.  ↓ *EcCaM1, EcCaML4, EcCaML14* and *EcCDPK14* in GP-45 at S2 and S4 stages. | qRT-PCR, RNAseq | Singh et al., 2014 |
|  |  |  |  |  |  |
| *EcCAX3, EcPM4ATPase8, EcPM12ATPase4, EcPM3ATPase,*  *EcER3ATPase3* | Calcium transport | HCaG: GP-45,  LCaG: GP-1 | **DS**  ↑ *EcPM4ATPase8*: S3 & S4 stages of GP-1 and S2, S3 & S4 stages of GP-45  ↑*EcPM12ATPase4*: S2, S3 & S4 stages of GP-1 and GP-45  ↑*EcPM3ATPase2*: S1, S3 and S4 stages of GP-1 and S2, S3 & S4 stages of GP-45  ↑*EcER3ATPase3:* S1 & S2 stages of GP-1 and S2 & S4 stages of GP-45  ↑*EcCAX3:* S1-S4stages of GP-1 and GP-45  ↓ *EcER3ATPase3:* S1 and S3 stages of GP-1 and GP-45  ↓*EcPM12ATPase4*: S1 stage of GP-1 and GP-45 | qRT-PCR,  RNAseq | Singh et al., 2015 |
|  |  |  |  |  |  |
| *EcZIP60* | Abiotic stress tolerance | GPU-28 | **VS**  ↑ leaves under 40 % drought FC, 300 mM NaCl, 10 µM ABA, 10 µM MV and PEG 6000 (-12b) at 3h and 6 h  ↓ leaves under 80% drought FC, 300 mM NaCl, 10 µM ABA, 10 µM MV and PEG 6000 (-12bars) at 1h and 12 h | RT-PCR, qRT-PCR | Babitha et al. 2015a |
|  |  |  |  |  |  |
| *EcbHLH57* | Abiotic stress tolerance | GPU-28 | **VS**  ↑leaves under 80% and 60% drought FC, 10µM ABA at 6 h and 12 h, PEG 6000 at 3 h and 6 h and 300 mM NaCl at 6h and 12h  ↓leaves under 40% drought FC, 10µM ABA at 1 h and 3 h, PEG 6000 at 1 h and 12 h and 300 mM NaCl at 1h and 3h | RT- PCR  qRT- PCR | Babitha et al. 2015b |
|  |  |  |  |  |  |
| *EcCIPK31-like* | Abiotic stress tolerance | GPU-28 | **VS**  ↑ shoots at 150 µM NaCl, 4°C cold, 42°C heat and rapid desiccation  ↓shoots at 10 µM MV  ↑roots at 150 µM NaCl, 4°C cold, 42°C heat, rapid desiccation and 10 µM MV  ↑leaves at 20 % and 40 % drought FC  ↓leaves at 60 % and 80 % drought FC | RT-PCR | Nagarjuna et al. 2016 |
|  |  |  |  |  |  |
| *EcNAC 67* | Salinity tolerance | Salinity susceptible: CO 12  Salinity tolerant: Trichy 1 | **VS**  ↑leaves and shoots of Trichy 1 at 300 mM NaCl  ↓roots of Trichy 1 at 300 mM NaCl | qRT-PCR | Rahman et al. 2016 |
|  |  |  |  |  |  |
| *EcPT1,*  *EcPT2,*  *EcPT3,*  *EcPT4* | Phosphate transport | Ragi Korchara Local, Khairna and VHC3611 | **VS**  **In the presence of AMF**  ↑*EcPT1*: roots and leaves of ragi korchara local and khairna at 30 DAS  ↓ *EcPT1*: roots and leaves of VHC3611 at 30 DAS  ↑*ECPT2*: roots of ragi korchara local, khairna, and VHC3611 at 30 DAS  ↑*ECPT3*: roots and leaves of ragi korchara local, khairna, and VHC3611 at 30 DAS  ↑*EcPT4*: roots of ragi korchara local, khairna, and VHC3611 at 30 DAS  **Under Pi stress**: **Ragi korchara local**  ↑*EcPT1*: roots and leaves at 6D  ↓ *EcPT1*: roots at 4D  ↑*EcPT2*: roots at 6D  ↓*EcPT2*: roots at 0D  ↑*EcPT3*: leaves at 4D and roots at 2D  ↓*EcPT3*: roots and leaves at 0D | RT-PCR, qRT-PCR | Pudake et al., 2017 |
|  |  |  |  |  |  |
| *EcCIPK24* | Calcium sensor | HCaG: GP-45,  LCaG: GP-1 | **VS**  ↑ roots, stems and leaves of GP-45 at 60 DAS  ↓ roots, stems and leaves of GP-1at 60 DAS  ↑ flag leaves of GP-45 at S1-S4 stages  ↓flag leaves of GP-1 at S4 stage  **DS**  ↑ GP-1 at S2 & S4 stages  ↑GP-45 at S1-S4 stage  ↓GP-1 at S1 and S3 stages | **RT-PCR, qRT-PCR** | Chinchole et al., 2017 |
|  |  |  |  |  |  |
| *EcGBF3*  *EcRGAP2*  *EcDBH*  *EcUN*  *EcHYP*  *EcRSLP* | Drought tolerance | GPU-28 | **VS**  ↑*EcUN, EcGBF3* and *EcHYP:* leaves  at 35% drought FC  ↑*EcDBH*: leaves at 35% drought FC recovery  ↑*EcRSLP*: leaves at 60% drought FC recovery  ↑*EcRGAP2*: leaves at 60% drought FC and 35% drought FC recovery  ↓*EcUN, EcGBF3* and *EcHYP*: leaves at 60% and 35% drought FC recovery  ↓*EcDBH, EcRGAP2* and *EcRSLP*: leaves at 35% drought FC recovery | qRT-PCR | Ramegowda et al. 2017 |
|  |  |  |  |  |  |
| *EcTAF6* | Abiotic stress tolerance | GPU-28  Drought tolerant:GE-1332  Drought susceptible: GE-156 | **VS**  ↑ leaves of GPU-28 at 60% drought FC, 300 mM NaCl, PEG  ↓leaves of GPU-48 at 40% and 20% drought FC and 10μM MV  ↑ leaves of GE-152 at 60% and 30% drought FC  ↓leaves of GE-152 at 100% drought FC  ↑ leaves of GE-1332 at 30% drought FC  ↓leaves of GE-1332 at 60 and 100% drought FC | RT-PCR, qRT-PCR | Parvathi and Nataraja 2017 |
|  |  |  |  |  |  |
| *Eco2, ECα-Prolamine* | Accumulation of prolamin, seed storage protein | HPG: GE-3885,  LPG: GE-1437 | **VS**  ↑*Eco2*: roots, stems and leaves of GE-3885 at 60 DAS  ↓ *Eco2*: roots, stems and leaves of GE-1437 at 60 DAS  **DS**  ↑ *Eco2*: GE3885 and GE-1437 at S2 stage  ↓ *Eco2*: GE3885 and GE-1437 at S1, S3 & S4 stage  ↑*ECα- Prolamine:* S1- S4 stages of GE3885 and S4 stage of GE-1437  ↓ *Eco2:* S1, S2 and S3 stage of GE-1437 | **RT-PCR, qRT-PCR** | Gaur et al., 2018 |
|  |  |  |  |  |  |
| *SGAT, GGAT, ICL, GLO, MHAR, APO, OXO* | Grain calcium accumulation | HCaG: GP-45,  LCaG: GP-1 | **DS**  ↑ *SGAT, GGAT, ICL, GLO, MHAR, APO* and *OXO*: S1, S3 and S4 stages of GP-1  ↓ *SGAT, GGAT, ICL, GLO, MHAR, APO* and *OXO*: S1, S3 and S4 stages of GP-45 | RT-PCR, qRT-PCR | Akbar et al., 2018 |
|  |  |  |  |  |  |
| *CAX1,*  *CAX3,*  *CaM,*  *CBL4,*  *CBL10,*  *CIPK24* | Calcium sensing and transport | HCaG: GP-45,  LCaG: GP-1 | **VS**  ↑*CAX1* and *CAX3*: roots of GP-45  ↓*CaM* and *Ca2+ ATPase*: roots of GP-1  ↑*CIPK24*: stems and leaves of GP-45 and GP-1  ↑*CaM* and *CAX1*: leaves of GP-45  **DS**  ↑*CAX1*:S1-S4 stages of GP-45 and GP-1  ↑*CaM* and *CAX3*: S1-S3 stages of GP-45 and GP-1  ↑*CIPK24*: S3 and S4 stages of GP-45 and GP-1  ↓*CaM* and *CAX3*: S4 stage of GP-45 and GP-1  ↓*CIPK24*: S1 and S2 stages of GP-45 and GP-1 | RT-PCR, qRT-PCR | Kokane et al., 2018 |

**^Abbreviations^**

*^AMF; Arbuscular mycorrhizae fungus, APO; Ascorbate peroxidase; ATPase^*^,^ *^adenine triphosphatase^*^; Ca; calcium;^ *^Cab, chlorophyll a/b binding protein^*^;^ *^CaM^*^,^ *^calmodulin^*^; CaML, CaM link protein;^ *^CaMK^*^, calmodulin dependent kinase;^ *^CAX^*^, cation exchanger;^ *^CBL^*^, Calciuneurin B-like protein;^ *^CDPK^*^, calmodulin^ *^independent protein kinases^*^, Conc, concentration;^ *^CIPK^*^,^ *^CBL-interacting protein kinase^*^; D; days; DAS; days after sowing;^ *^Dof^*^,^ *^DNA binding with one finger^*^; DS, developing spikes;^ *^EcbHLH57^*^,^ *^Eleusine coracana^* ^basic helix-loop-helix;^ *^EcCIPK31^*^-like,^ *^CBL Interacting Protein Kinase^*^;^ *^EcDBH, Eleusine coracana^* *^DEAD/DEAH box helicase^*^;^ *^EcGBF3, Eleusine coracana^* *^G-Box binding factor 3^*^;^ *^EcHYP, Eleusine coracana^* *^hypothetical protein^*^;^ *^EcRGAP2, Eleusine coracana^* *^Rho GTPase activating protein 2^*^;^ *^EcRSLP, Eleusine coracana^* *^RNase S-like protein precursor^*^;^ *^EcUN, Eleusine coracana^* *^unknown protein^*^;^ *^EcPT^*^,^ *^Eleusine coracana^* *^phosphate transporter^*^;^ *^EcTAF6^*^,^ *^TATA-box binding protein associated factor6^*^;^ *^EcNAC^*^, NAM, ATAF1-2, and CUC2;^ *^EcNR^*^,^ *^Eleusine coracana^* *^nitrate^* *^reductase^*^,^ *^EcGS^*^,^ *^Eleusine coracana^* *^glutamine synthetase^*^;^ *^Eco2^*^,^ *^Eleusine coracana OPAQUE2; EcZIP60, Eleusine coracana basic leucine zippers; FC, field capacity; GGAT^*^,^ *^glutamate glyoxylate amino-transferase^*^;^ *^GLO, glyoxal oxidase^*^;^ *^GOGAT^*^,^ *^glutamine oxoglutarate aminotransferase^*^; HCaG, high calcium genotype;^ *^HNRT, high-affinity nitrate transporter^*^; HPG, high protein genotype;^ *^ICL^*^,^ *^isocitrate lyase^*^; LCaG, low calcium genotype; LPG, low protein genotype;^ *^LNRT^*^,^ *^low affinity nitrate transporter^*^;^ *^ME^*^,^ *^malic enzyme^*^;^ *^MHAR^*^,^ *^Mono dehydro ascorbate reductase^*^; MPG, medium protein genotype^*^;^* ^MV, methyl viologen;^ ^N, Nitrogen^ *^OXO^*^, NaCl, sodium chloride;^ *^oxalate oxidase^*^;^ *^PEPC^*^,^ *^Phosphoenol pyruvate carboxylase^*^;^ *^PEPC-k^* ^,^ *^phosphoenol pyruvate carboxykinase^*^;^ *^PK^*^,^ *^pyruvate kinase^*^;^ *^PPDK^*^,^ *^pyruvate dikinase^*^;^ *^PBF, prolamin-binding factor^*^; qRT-PCR, quantitative real time polymerase chain reaction;^ *^RBCS^*^, rubisco; RT-PCR, reverse transcriptase polymerase chain reaction; RNAseq, RNA sequencing; S1, spike emergence stage; S2, pollination stage; S3; dough stage; S4, maturation stage;^ *^SGAT^*^,^ *^serine glyoxylate amino-transferase^*^;^ *^SnRK1^*^,^ *^sensor protein kinase^*^;^ *^SPS^*^,^ *^sucrose phosphate^* *^synthase^*^;^ *^TPC1^* ^-^ *^two pore channel1^*^; VS, vegetative stage; ↑, gene up-regulation; ↓, gene down regulation^

**References**

Akbar, N., Gupta, S., Tiwari, A., Singh, K., and Kumar, A. (2018). Characterization of metabolic network of oxalic acid biosynthesis through RNA seq data analysis of developing spikes of finger millet (*Eleusine coracana*): Deciphering the role of key genes involved in oxalate formation in relation to grain calcium accumulation. *Gene* 649**,** 40-49.

Babitha, K., Ramu, S., Nataraja, K.N., Sheshshayee, M., and Udayakumar, M. (2015a). EcbZIP60, a basic leucine zipper transcription factor from *Eleusine coracana* L. improves abiotic stress tolerance in tobacco by activating unfolded protein response pathway. *Mol. Breed.* 35, 181-197.

Babitha, K., Vemanna, R.S., Nataraja, K.N., and Udayakumar, M. (2015b). Overexpression of EcbHLH57 transcription factor from *Eleusine coracana* L. in tobacco confers tolerance to salt, oxidative and drought stress. *PLoS ONE* 10, e0137098.

Chinchole, M., Pathak, R.K., Singh, U.M., and Kumar, A. (2017). Molecular characterization of EcCIPK24 gene of finger millet (*Eleusine coracana*) for investigating its regulatory role in calcium transport. *3Biotech* 7**,** 267. doi: 10.1007/s13205-017-0874-7

Gaur, V.S., Kumar, L., Gupta, S., Jaiswal, J., Pandey, D., and Kumar, A. (2018). Identification and characterization of finger millet OPAQUE2 transcription factor gene under different nitrogen inputs for understanding their role during accumulation of prolamin seed storage protein. *3Biotech* 8**,** 163. doi: 10.1007/s13205-018-1150-1

Gupta, A.K., Gaur, V.S., Gupta, S., and Kumar, A. (2013). Nitrate signals determine the sensing of nitrogen through differential expression of genes involved in nitrogen uptake and assimilation in finger millet. *Funct. Integ. Genom.* 13**,** 179-190.

Gupta, N., Gupta, A.K., Singh, N., and Kumar, A. (2011). Differential expression of PBF Dof transcription factor in different tissues of three finger millet genotypes differing in seed protein content and color. *Plant Molec. Biol. Rep.* 29**,** 69-76.

Gupta, S., Gupta, S.M., Gupta, A.K., Gaur, V.S., and Kumar, A. (2014). Fluctuation of Dof1/Dof2 expression ratio under the influence of varying nitrogen and light conditions: involvement in differential regulation of nitrogen metabolism in two genotypes of finger millet (*Eleusine coracana* L.). *Gene* 546**,** 327-335.

Kanwal, P., Gupta, S., Arora, S., and Kumar, A. (2014). Identification of genes involved in carbon metabolism from *Eleusine coracana* (L.) for understanding their light-mediated entrainment and regulation. *Plant Cell Rep.* 33**,** 1403-1411.

Kokane, S., Pathak, R., Singh, M., and Kumar, A. (2018). The role of tripartite interaction of calcium sensors and transporters in the accumulation of calcium in finger millet grain. *Biologia Plant.* 1-10.

Mirza, N., Taj, G., Arora, S., and Kumar, A. (2014). Transcriptional expression analysis of genes involved in regulation of calcium translocation and storage in finger millet (*Eleusine coracana* L. Gartn.). *Gene* 550**,** 171-179.

Nagarjuna, K., Parvathi, M., Sajeevan, R., Pruthvi, V., Mamrutha, H., and Nataraja, K. (2016). Full-length cloning and characterization of abiotic stress responsive CIPK31-like gene from finger millet, a drought-tolerant crop. *Curr. Sci.* 111, 890-896.

Parvathi, M., and Nataraja, K.N. (2017). Discovery of stress responsive TATA-box binding protein associated Factor6 (TAF6) from finger millet (*Eleusine coracana* (L.) Gaertn). *J. Plant Biol*. 60, 335-342.

Pudake, R.N., Mehta, C.M., Mohanta, T.K., Sharma, S., Varma, A., and Sharma, A.K. (2017). Expression of four phosphate transporter genes from Finger millet (*Eleusine coracana* L.) in response to mycorrhizal colonization and Pi stress. *3Biotech* 7**,** 17. doi: 10.1007/s13205-017-0609-9

Rahman, H., Ramanathan, V., Nallathambi, J., Duraialagaraja, S., and Muthurajan, R. (2016). Over-expression of a NAC 67 transcription factor from finger millet (*Eleusine coracana* L.) confers tolerance against salinity and drought stress in rice. *BMC Biotechnol.* 16, 35-49.

Ramegowda, V., Senthil-Kumar, M., Nataraja, K.N., Reddy, M.K., Mysore, K.S., and Udayakumar, M. (2012). Expression of a finger millet transcription factor, EcNAC1, in tobacco confers abiotic stress-tolerance. *PloS ONE*. 7, e40397.

Ramegowda, V., Gill, U.S., Sivalingam, P.N., Gupta, A., Gupta, C., Govind, G., Nataraja, K.N., Pereira, A., Udayakumar, M., and Mysore, K.S. (2017). GBF3 transcription factor imparts drought tolerance in *Arabidopsis thaliana*. *Sci. Rep.* 7, 9148-9161.

Singh, U.M., Chandra, M., Shankhdhar, S.C., and Kumar, A. (2014). Transcriptome wide identification and validation of calcium sensor gene family in the developing spikes of finger millet genotypes for elucidating its role in grain calcium accumulation. *PloS One* 9**,** e103963. doi.org/10.1371/journal.pone.0103963

Singh, U.M., Metwal, M., Singh, M., Taj, G., and Kumar, A. (2015). Identification and characterization of calcium transporter gene family in finger millet in relation to grain calcium content. *Gene* 566**,** 37-46.
